# Synthesis and Properties of Sulfur-Containing Organophosphorus Extractants Based on Red Phosphorus, Alkyl Bromides, and Elemental Sulfur

**DOI:** 10.3390/ma16093394

**Published:** 2023-04-26

**Authors:** Gaukhar K. Bishimbayeva, Nina K. Gusarova, Arailym M. Nalibayeva, Svetlana I. Verkhoturova, Amangul Bold, Natalya A. Chernysheva, Assem K. Zhangabayeva, Svetlana N. Arbuzova, Yerlan N. Abdikalykov, Dinara S. Zhumabayeva

**Affiliations:** 1 D.V. Sokolsky Institute of Fuel, Catalysis and Electrochemistry, Kunayev, 142, Almaty 050010, Kazakhstan; 2 A.E. Favorsky Irkutsk Institute of Chemistry, Siberian Branch Russian Academy of Sciences, St. Favorskogo, 1, 664033 Irkutsk, Russia

**Keywords:** organic phosphine sulfides, organic phosphine oxides, red phosphorus, sulfur, synthesis, metal extractants

## Abstract

In order to obtain sulfur-containing organophosphorus compounds that are promising as extractants of heavy metals, the interaction of elemental phosphorus and sulfur with alkyl bromides catalyzed using strong bases was studied. According to the task, the reaction of non-toxic and non-flammable red phosphorus with alkyl bromides under conditions of phase transfer catalysts (PTC), followed by the introduction of elemental sulfur into the reaction medium, were studied. It is shown that alkyl bromides interact with red phosphorus when heated (95–105 °C, 5–8 h) under conditions of phase transfer catalysts (PTC) in a two-phase system: a 60% aqueous solution of KOH-toluene-benzyltriethylammonium chloride (BTEAC) forming a mixture of organophosphorus compounds along with alkylphosphines (57–60%), are the main reaction products; alkylphosphine oxides are also formed (40–43%). The introduction of elemental sulfur (solution in toluene) at the final stage of the process into the reaction mass cooled to 40–60 °C leads to the expected alkylphosphine sulfides, which are the result of the interaction of alkylphosphines with sulfur. The formation of complex mixtures of products prevents the release of target alkylphosphine sulfides in individual form. However, the synthesized mixture of alkylphosphine sulfides and alkylphosphine oxides without separation into individual components is promising for studying its extraction properties in relation to heavy metals. Testing of the extraction properties of synthesized mixtures of alkylphosphine sulfides and alkylphosphine oxides in relation to heavy metals (Ni, Co, Zn, Pb) and noble metals (Ag) showed that the resulting mixtures of tertiary phosphine oxides and phosphine sulfides are highly effective extractants. The degree of extraction in relation to Ni, Co, Zn, and Pb varies from 99.90 to 99.99%, and for Ag from 99.56 to 99.59%.

## 1. Introduction

Organic phosphorus compounds are actively studied by leading scientists around the world. This is due to the high reactivity of these compounds and their wide range of practical and useful properties. Sulfur-containing organophosphorus compounds are widely used in engineering, agriculture, and medicine as corrosion protection and salt deposits in industrial waters; stabilizers and plasticizers of polymers; monomers for ion-exchange and thermostable polymers; additives for lubricating oils and hydraulic fluids [1,2,3,4,5]; and complexons and extractants in the isolation of precious, non-ferrous, and heavy metals in hydrometallurgical processes [6,7,8,9]. Extraction methods for the separation and concentration of metals are the most productive. They are widely implemented in hydrometallurgy because it needs to search for and create new effective extractants [10,11]. Among the compounds suitable for use as extractants, organic phosphine sulfides and phosphine oxides hold a special place: they allow extraction processes to be carried out with good selectivity and efficiency [12,13,14,15,16,17,18,19,20,21]. Currently, extractants based on various phosphine sulfides and phosphine oxides under the commercial name CYANEX are widely known in the hydrometallurgical industry. For example, the commercial extractant Cyanex 471x with the active component tri-isobutyl phosphine sulfide (TIBPS) is widely used in the extraction and separation of valuable metals, such as palladium [22,23], gold [23,24], silver [25,26], cadmium [27,28], and mercury [29,30,31]. Another extractant of this brand, CYANEX-923, is a mixture of trialkylphosphine oxides with a C6-C8 hydrocarbon of radical length [32,33]. Phosphine oxides are the most effective extractants among neutral organic compounds; this is confirmed by a large amount of literature data on the successful use of trioctylphosphine oxide (TOPO) as an extractant in the processes of extraction and separation of valuable metals, including rare (RM) and rare earths (REM) [34,35]. It should be noted that extractants, which are most widely used in industry and presented in the literature, contain donor centers of one specific type: hard (oxygen) or soft (sulfur). Ligands containing groups with hard centers (most often in the composition of phosphine oxide fragments) demonstrate high efficiency of binding with heavy metal ions but have low selectivity [32,33,36,37]; reagents with soft centers (sulfur atom), to the contrary, are characterized by high selectivity, but their use is complicated by low efficiency [22,23,24,25,26,27,28,29,30,31,37,38].

Far from the methods of “green” chemistry, the production of phosphine sulfides and phosphine oxides is mainly based on the use of labor-intensive multi-stage approaches that use environmentally unsound phosphorus halides or toxic and flammable white phosphorus [39]. Therefore, the development of resource-saving and safer technological approaches for the synthesis of organophosphorus compounds based on available and man-made raw materials to create highly effective extractants of heavy metals is an urgent area of scientific research which will solve important problems in the field of hydrometallurgy and extraction chemistry.

In our research, we decided to combine two different donor binding centers in one extractant in order to achieve their synergistic effect: the target compounds are mixtures of O- and S-donor alkylphosphine oxides and alkylphosphine sulfides. This paper presents the results of studying the reaction of alkyl bromides with red phosphorus, which is nontoxic and less flammable when compared to the white allotropic modification of phosphorus. This was completed under conditions of phase transfer catalysts (PTC) with subsequent introduction of elemental sulfur into the reaction medium which is capable of producing alkylphosphine oxides and alkylphosphine sulfides. Obtaining extractants in the form of a mixture is an advantage of the one spot methodology proposed by us, since the synthesized mixtures of alkylphosphine oxides and sulfides without separation into individual components are highly effective extractants in relation to heavy and noble metals. It should be noted that we used elemental sulfur, which is a technogenic raw material, i.e., a waste of desulfurization of the oil and gas chemical industry.

The study was carried out as part of the development of a new methodology for the synthesis of organophosphorus compounds based on red phosphorus without the use of toxic, self-igniting, white phosphorus and environmentally hazardous phosphorus halides and oxyhalides [40,41].

## 2. Materials and Methods

### 2.1. Reagents Used

#### 2.1.1. Reagents for the Synthesis of Extractants

Phosphorus red (≥97.0%, Sigma-Aldrich, St. Louis, MI, USA), 1-Bromoheptane (99.0%, Sigma-Aldrich), 1-Bromooctane (99.0%, Sigma-Aldrich), toluene (≥99.7%, Sigma-Aldrich), potassium hydroxide (ACS reagent, ≥85%), BTEAC—Benzyltriethylammonium chloride (99.0%, Sigma-Aldrich), and granulated sulfur-product of desulfurization of Tengiz crude oil (Table 1). 

Distilled water was used for all the experiments.

All synthetic experiments were carried out in a dry, inert atmosphere (argon).

#### 2.1.2. Extraction Materials

Mixtures of heptylphosphine oxides and heptylphosphine sulfides and of octylphosphine oxides and octylphosphine sulfides were used as extractants. 

These mixtures were synthesized according to the methodology below. 

Extraction properties of the synthesized extractants (mixtures of alkylphosphine sulfides and alkylphosphine oxides) in relation to metals (Co^2+^, Pb^2+^, Zn^2+^, Ni^2+^, Ag^+^) were studied in model solutions with a metal concentration of 5000 mg/dm^3^, a weight of extractant of 2 g, a weight of hydrocarbon diluent of 18 g (10% by mass), a volume of the model solution of 200 mL, a contact time of 20 min, a pH of model solutions of 2–3, and a temperature of 24 °C (the ambient temperature).

Aviation kerosene produced by JSC “Pavlodar Petrochemical Plant” (Pavlodar, Kazakhstan) was used as a diluent: SF-1 of the highest grade with a density of 0.776 g/cm^3^.

The model solutions with the content of the studied metal ions 5000 mg/dm^3^ (Co^2+^, Pb^2+^, Zn^2+^, Ni^2+^, Ag^+^) were prepared by dissolving precise attachments of the corresponding salts (Silver(I) nitrate AgNO_3_, Lead(II) nitrate Pb(NO_3_)_2_, Nickel(II) sulfate heptahydrate NiSO_4_·7H_2_O, Cobalt(II) sulfate heptahydrate CoSO_4_·7H_2_O, Zinc(II) sulfate heptahydrate ZnSO_4_·7H_2_O) in distilled water. The acidity of the model solutions was monitored using a pH-150 MI meter.

### 2.2. Equipment and Measurement Methods

The IR spectra of the synthesized mixtures of alkylphosphine sulfides and alkylphosphine oxides were recorded on a Varian 3100 FT-IR spectrometer (Varian, Walnut Creek, CA, USA) in a thin layer (Supplementary Materials).

IR spectra of the organic phase after extraction of the silver and lead with a mixture of octylphosphine oxides and octylphosphine sulfides in a diluent (kerosene) were recorded on a Nicolet iS5 spectrometer (Thermo Scientific, Waltham, MA, USA) in a thin layer.

The NMR spectra of ^1^H, ^13^C, and ^31^P (400.13, 100.62, and 161.98 MHz, respectively) were obtained using Bruker DPX 400 (Bruker, Karlsruhe, Germany) and Bruker AV-400 (Bruker, Karlsruhe, Germany) spectrometers; the internal standard is HMDS (^1^H) and the external standard is 85% H_3_PO_4_ (^31^P). When describing NMR spectra, the following abbreviations are used: s—singlet, d—doublet, t—triplet, m—multiplet. 

The electronic absorption spectra of the solutions were recorded on a PE-5400UV (EKROS, St. Petersburg, Russia) spectrophotometer in the visible region. The mass concentrations of cobalt, zinc, and nickel ions were determined by photometric method [42,43,44].

The atomic absorption analysis of solutions containing silver ions was carried out on a Varian AA240 (Agilent Technologies, Santa Clara, CA, USA) spectrometer. 

The atomic emission analysis of solutions containing lead and lanthanum ions was carried out on a Perkin Elmer Optima 8300 DV ICP-OES (Perkin Elmer, Waltham, MA, USA) inductively coupled plasma spectrometer.

The elemental analysis was performed using the Flash EA 1112 CHNS analyzer (Thermo Scientific, USA).

### 2.3. Extraction Procedure

The extraction was carried out by simultaneous contact of the extractant solution in a diluent and the aqueous solutions of the salts of the metals under study with constant stirring on a magnetic stirrer. The room temperature was 24 °C and the contact time was 20 min, which ensured the achievement of equilibrium in the system. The ratio of organic and aqueous phases in the study of extraction properties is 1:10. At the end of the extraction, the organic layer was separated using a dividing funnel, and the concentration of metal ions was determined in the residual salt solution. The metal content in the organic phase was calculated from the difference between the concentrations of metals before and after the extraction.

The degree of substrate extraction (E, %) was calculated from the residual amount of metal in the aqueous phase from the ratio:

$$E\%=\frac{\left(\mathrm{Co}-\mathrm{Ck}\right)}{\mathrm{Co}}\;100,$$

where Co is the concentration of metal in the initial solution, and Ck is the residual concentration of metal in the solution after extraction.

### 2.4. Synthesis of Extractants

**Scheme 1:**
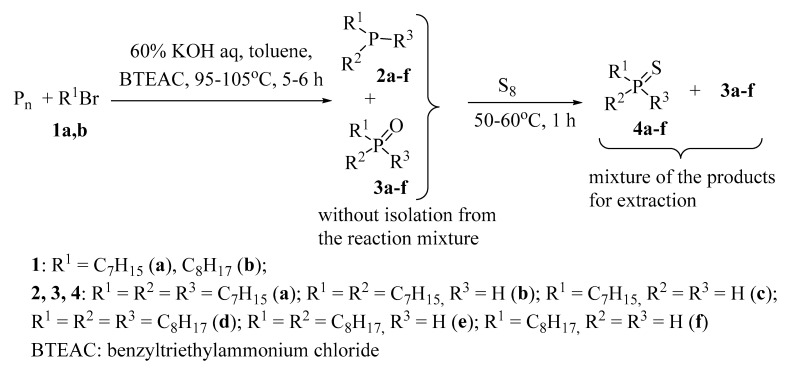
Synthesis of extractants (mixtures of compounds **3a**–**f** and **4a**–**f**).

Extractants were obtained by the interaction of red phosphorus with alkyl bromides **1a**,**b** in the presence of phase transfer catalysts (PTC), followed by the introduction of elemental sulfur into a reaction mixture containing alkylphosphines **2a**–**f** and alkylphosphine oxides **3a**–**f** formed at the first stage (Scheme 1). 

#### Methods of Synthesis of Extractants

Synthesis of a mixture of heptylphosphine oxides 3a,b and heptylphosphine sulfides **4a**,**b**. Red phosphorus (6.2 g, 0.2 mol), toluene (90 mL), heptyl bromide **1a** (17.01 g, 0.1 mol), and BTEAC (1.14 g, 5 mol%) were placed in a three-neck flask equipped with a mechanical stirrer, a refrigerator, and a thermometer. Argon was bubbled through the reaction mixture. Then, with a little stirring, a freshly prepared 60% aqueous solution of KOH (60 g KOH in 40 mL of water) was carefully added while heating the reaction mixture to 60 °C. The reaction mixture was stirred at 105–110 °C (glycerin bath) for 5 h. The reaction mass was cooled to 50 °C, analyzed (according to NMR ^31^P, signals of heptylphosphines **2a**–**c** and heptylphosphine oxides **3a**–**c** are present in the reaction mixture in a ratio of ~1:0.8), and then a sulfur solution (0.7 g, 0.02 mol) was added drop by drop in 30 mL of toluene; a color change from intense yellow to light yellow was observed. The reaction mixture was stirred for another 1 h, left for 12 h, analyzed (according to NMR ^31^P, signals of heptylphosphine oxides **3a**–**c** and heptylphosphine sulfides **4a**–**c** are present in the reaction mixture in a ratio of ~0.8:1), diluted with water (180 mL), and the upper organic layer separated, washed with water (3 × 30 mL), dried over calcined potash, and analyzed (according to NMR ^31^P, signals of heptylphosphine oxides **3a**,**b** and heptylphosphine sulfides **4a**,**b** are present in the reaction mixture in a ratio of ~0.9:1, signals of primary heptylphosphine oxide **3c** and sulfide **4c** were not observed). Toluene was distilled at reduced pressure. The unreacted heptyl bromide was then distilled off from the residue with a water jet pump (69% conversion). The residue was analyzed by physico-chemical methods. A quantity of 4.42 g (52%) of a mixture of heptylphosphine oxides **3a**,**b** and heptylphosphine sulfides **4a**,**b** was received. Found, %: C 59.00, H 9.50, P 8.7, S 5.59. 

Synthesis of a mixture of **3d**,**e** octylphosphine oxides and **4d**,**e** octylphosphine sulfides. This was synthesized similarly to the previous one from (3.1 g, 0.1 mol) red phosphorus and (9.66 g, 0.05 mol) octyl bromide **1b** with the addition of (0.57 g, 5 mol%) BTEAC. At the same time, the volumes of solvents and solutions of all other reagents were reduced by a factor of two. In this experiment, octyl bromide was not distilled off; its conversion was ~100%. A quantity of 3.25 g (47%) of a mixture of **3d**,**e** octylphosphine oxides and **4d**,**e** octylphosphine sulfides was obtained (according to NMR ^31^P, signals of octylphosphines oxides **3d**,**e** and octylphosphine sulfides **4d**,**e** are present in the reaction mixture in a ratio of ~0.9:1); Found, %: C 62.29; H 10.13; P 6.68; S 2.32.

The production of an enlarged batch of extractants for further studies of their extraction activity was carried out by scaling the synthesis described above tenfold. The yields and compositions of the target mixtures did not change fundamentally as a result of the scaled synthesis.

The formation of compounds **2** and **3** was proved by studying the composition of the reaction mixture obtained before the addition of sulfur; the formation of compounds **4c**,**f** was proved by studying the composition of the reaction mixture obtained before the treatment with water.

### 2.5. Spectral and Physicochemical Characteristics of Compounds

#### 2.5.1. Spectral Characteristics of Heptylphosphines **2**, Heptylphosphine Oxides **3**, and Heptylphosphine Sulfides **4**

Compounds **2a**, **3a**–**c**, **4a**,**b** were identified using known samples; the spectral characteristics of compounds **2b**,**c** are identical to the ones in the literature [45].

Triheptylphosphine **2a**. NMR spectrum ^31^P (toluene): δ_P_ −31.3 ppm, s. 

Diheptylphosphine **2b**. NMR spectrum ^31^P: δ_P_ −68.3 ppm, d, *J*_PH_ = 195 Hz (toluene); literature data: −68.8 ppm, *J*_PH_ = 196 (CDCl_3_) [45].

Heptylphosphine **2c**. NMR spectrum ^31^P: δ_P_ −137.5 ppm, t, *J*_PH_ = 190 Hz (toluene). literature data: −137.1 ppm, t, ^1^*J*_PH_ 193 Hz (CDCl_3_) [45].

Triheptylphosphine oxide **3a**. NMR spectrum ^31^P: δ_P_ 43.3 ppm, (toluene), δ_P_ 49.4 ppm (CDCl_3_), s.

Diheptylphosphine oxide **3b**. NMR spectrum ^31^P: δ_P_ 29.8 ppm, d (toluene), δ_P_ 28.8 ppm (CDCl_3_), d, *J*_PH_ = 456 Hz. 

Heptylphosphine oxide **3c**. NMR spectrum ^31^P (toluene): δ_P_ 12.7 ppm, t, *J*_PH_ = 424 Hz. 

Triheptylphosphine sulfide **4a**. NMR spectrum ^31^P (CDCl_3_): δ_P_ 49.2 ppm, s.

Diheptylphosphine sulfide **4b**. NMR spectrum ^31^P: (CDCl_3_): δ_P_ 28.6 ppm, d, *J*_PH_ = 454 Hz. 

Mixture of heptylphosphine oxides **3a**,**b** and heptylphosphine sulfides **4a**,**b**. IR spectrum, ν, cm^−1^: 3359 m, 3061 w, 3027 w, 2955 s, 2926 s, 2855 s, 2730 w, 2675 w, 2280 m, 1732 w, 1653 w, 1602 w, 1494 w, 1462 s, 1406 w, 1378 m, 1345 w, 1300 w, 1272 w, 1272 w, 1246 w, 1230 w, 1200 m, 1170 s, 1113 w, 1054 m, 1029 w, 958 w, 928 w, 896 w, 816 w, 773 w, 754 w, 723 m, 700 w, 614 m, 561 w, 488 m.

#### 2.5.2. Spectral and Physicochemical Characteristics of Octylphosphines **2**, Octylphosphine Oxides **3**, and Octylphosphine Sulfides **4**

Compound **4e** was identified using known samples; the spectral characteristics of compounds **2d**–**f**, **3d**–**f**, **4d** are identical to the ones in the literature [45,46,47,48,49,50].

Trioctylphosphine **2d**. NMR spectrum ^31^P: δ_P_ −31.4 ppm, s (toluene); literature data: δ_P_ −31.8 ppm, s (CDCl_3_) [48]. 

Dioctylphosphine **2e**. NMR spectrum ^31^P: δ_P_ −68.4 ppm, d, *J*_PH_ = 186 Hz (toluene); literature data: −69.1 ppm, d, *J*_PH_ = 189 Hz (CDCl_3_) [49].

Octylphosphine **2f**. NMR spectrum ^31^P: δ_P_ −137.6 ppm, t, *J*_PH_ = 190 Hz (toluene); literature data: −137.1 ppm, t, ^1^*J*_PH_ 190 Hz [45,47]. 

Trioctylphosphine oxide **3d**. NMR spectrum ^1^H (CDCl_3_), δ: 0.84 t (9 H, ^3^*J*_HH_ = 7.1 Hz, CH_3_), 1.19–1.30 m (24 H, (CH_2_)_4_-Me), 1.33–1.43 m (6 H, CH_2_-Am), 1.48–1.53 m (6 H, CH_2_-Hex), 1.63–1.81 m (6 H, CH_2_-P). NMR spectrum ^31^P: δ_P_ 43.9 ppm, s (toluene); δ_P_ 50.7 ppm, s (CDCl_3_); literature data: δ_P_ 48.9 ppm, s (CDCl_3_) [46]. Anal. Calcd (%) for C_24_H_51_OP: C, 74.56; H, 13.30; P, 8.01. Found: C, 74.85; H, 13.18; P, 7.89.

Dioctylphosphine oxide **3e**. NMR spectrum ^1^H (CDCl_3_), δ: 0.86 t (6 H, ^3^*J*_HH_ = 7.0 Hz, CH_3_), 1.20–1.33 m (16 H, (CH_2_)_4_-Me), 1.34–1.44 m (4 H, CH_2_-Am), 1.52–1.66 m (4 H, CH_2_-Hex), 1.69–1.84 m (4 H, CH_2_-P), 6.83 d (1 H, PH, ^1^*J*_HP_ = 438 Hz. NMR spectrum ^13^C (CDCl_3_), δ: 14.0 (C^8^, Me), 21.7 (C^2^), 22.6 (C^7^), 28.3 d (C^1^, CH_2_-P, ^1^*J*_PC_ = 65.0 Hz), 28.96 and 29.04 (C^4,5^), 30.7 d (C^3^, ^3^*J*_PC_ = 13.6 Hz), 31.7 (C^6^). NMR spectrum ^31^P: δ_P_ 30.1 ppm (toluene), δ_P_ 35.4 ppm (CDCl_3_), d, *J*_PH_ = 435 Hz; literature data: δ_P_ 28.0 ppm [48], 35.0 ppm, d, *J*_PH_ = 435 Hz (CDCl_3_) [46,49]. Anal. Calcd (%) for C_16_H_34_OP: C, 70.03; H, 12.86; P, 11.29. Found: C, 70.25; H, 12.98; P, 11.02.

Octylphosphine oxide **3f**. NMR spectrum ^31^P: δ_P_ 12.7 ppm, t, *J*_PH_ = 458 Hz (toluene); literature data: δ_P_ 10 ppm, t, ^1^*J*_PH_ 464 Hz [47].

Trioctylphosphine sulfide **4d**. NMR spectrum ^31^P: δ_P_ 49.5 ppm, s (CDCl_3_); literature data: 49.3 ppm [50].

Dioctylphosphine sulfide **4e**. NMR spectrum ^31^P: δ_P_ 33.4 ppm, d, *J*_PH_ = 454 Hz.

Mixture of octylphosphine oxides 3d,e and octylphosphine sulfides **4d**,**e**, IR spectrum, ν, cm^−1^: 3363 w, 2953 s, 2923 s, 2853 s, 2730 w, 2728 w, 2687 w, 2655 w, 2572 w, 2492 w, 2323 w, 1653 w, 1541 w, 1463 s, 1408 w, 1378 w, 1239 w, 1197 w, 1173 m, 1118 w, 1024 m, 987 w, 972 s, 852 w, 804 w, 754 m, 720 w, 702 w, 679 w, 605 w, 516 w, 461 w.

#### 2.5.3. Spectral Characteristics of the Organic Phases after Extraction

The organic phase after silver extraction with a mixture of octylphosphine oxides and octylphosphine sulfides in kerosene. IR spectrum, ν, cm^−1^: 3176 w; 2956 s, 2923 s, 2874 s, 2858 s, 2731 w, 2667 w, 1606 w, 1460 m, 1382 m, 1340 m, 1301 w, 1173 m, 1158 w, 1028 w, 970 w, 915 w, 885 w, 849 w, 808 w, 785 w, 769w, 743 m, 726 w, 704 w, 548 w, 431 w.

The organic phase after extraction of lead with a mixture of octylphosphine oxides and octylphosphine sulfides in kerosene. IR spectrum, ν, cm^−1^: 3173 w; 2955 s, 2923 s, 2874 s, 2855 s, 2725 w, 2667 w, 1609 w, 1463 m, 1382 m, 1340 m, 1304 w, 1184 w, 963 w, 892 w, 846 w, 811 w, 785 w, 766 w, 746 m, 723 w, 697 w, 555 w.

## 3. Results and Discussion

### 3.1. Extractant Preparation

It is shown that hexyl- and octyl bromides **1a**,**b** interact with red phosphorus when heated (105–110 °C, 5–6 h, argon atmosphere) under PTC conditions: 60% aqueous solution of KOH-toluene-benzyltriethylammonium chloride (BTEAC) forms mixtures of organophosphorus compounds; the corresponding alkylphosphine oxides **3a**–**f** were also formed (40–43%, NMR ^31^P data) along with alkylphosphines **2a**–**f**—the main reaction products (57–60% according to NMR ^31^P). The introduction of elemental sulfur (solution in toluene) at the final stage of the process into the reaction mass further leads to the formation of alkylphosphine sulfides **4a**–**f**, which are the result of the interaction of the alkylphosphines present in the reaction medium with sulfur (Scheme 1).

As can be seen from the data in Table 2, carrying out the reaction (Scheme 1) at a molar ratio of the reagents P_n_:AlkBr = 1:0.5 and a temperature of 105–110 °C for 5–6 h achieves a complete conversion of red phosphorus; the conversion of alkyl bromide is 69–100% (Table 2, ex. 1–3). A decrease in the reaction temperature to 90–94 °C reduces the conversion of red phosphorus to 80% and alkyl bromide to 24% (Table 2, ex. 4). An increase in the amount of the initial alkyl bromide also leads to a decrease in the conversion of the initial reagents (Table 2 ex. 4–6). In this case, a decrease in the amount of the initial alkyl bromide is impractical despite the quantitative conversion of the initial reagents as the yield of target products **3**, **4** sharply decreases (more than twice) (Table 2 ex. 7).

It should be noted that the ratio of P_n_:AlkBr reagents has a significant effect on the ratio of the formed primary, secondary, and tertiary alkylphosphines **2** and their corresponding phosphine oxides **3** (NMR ^31^P data, spectra are given in Section 2.5). Thus, in the case of the molar ratio of the reagents P_n_:AlkBr = 1:0.5 (Table 2, ex. 1, 2), tertiary products **2a**,**d** and **3a**,**d** predominate, while primary and secondary phosphines **2b**,**c**,**e**,**f** and phosphine oxides **3b**,**c**,**e**,**f** are also formed but in significant quantities.

Using the reaction of red phosphorus with octyl bromide as an example, it has been shown that the content of tertiary phosphines and phosphine oxides increases with the amount of the initial alkyl bromide (Table 2, ex. 3–6). At the molar ratio of the reagents, P_n_:OctBr = 1:1.175 trioctylphosphine **2d** and trioctylphosphine oxide **4d** become almost the only products (Table 2, ex. 6). At the same time, a decrease in the amount of the initial alkyl bromide leads to an increase in the content of secondary and primary phosphines **2e**,**f** and phosphine oxides **3e**,**f** (Table 2, ex. 7).

It is shown that an increase in the reaction time to 8 h was accompanied by complete oxidation of the intermediate phosphines **2** into phosphine oxides **3** due to interaction with air oxygen, which apparently enters the reaction mixture due to the additional reaction time as well as the additional amount of sampling for analysis. As a result, in this case it was not possible to obtain the corresponding phosphine sulfides 4 (Table 2, ex. 5).

Thus, the conducted studies allow us to recommend several conditions for the preparative synthesis method of alkylphosphines **2** and their corresponding phosphine oxides **3** from red phosphorus and alkyl bromides **1** under the conditions of the PTC (Scheme 1): 60% aqueous solution of KOH-toluene-BTEAC, 105–110 °C, 5–6 h, reagent ratio P_n_:AlkBr = 1:0.5, and inert atmosphere (argon). The interaction of alkylphosphines **2** with the elemental sulfur introduced into the reaction mass cooled to 40–60 °C at the second stage of the reaction (Scheme 1) leads to a complete conversion of phosphines **2**, which are quantitatively converted into the corresponding alkylphosphine sulfides **4**. 

In addition, a detailed analysis of the reaction mixtures showed that the reaction results in the formation (in small quantities) of alkylphosphonic and phosphinic acids as well as their sulfur-containing analogues. Thus, signals in the region of 51–53 ppm (^31^P NMR, toluene) can be attributed to dialkyl phosphinic acids (Alk)_2_P(O)OH and the corresponding alkyl esters (Alk)_2_P(O)OAlk; signals in the region of 76–77 ppm (NMR ^31^P, CDCl_3_) to the dialkyl thiophosphinic acids (Alk)_2_P(S)OH; signals in the region of 47–48 ppm, *J*_PH_ = 540 Hz (NMR ^31^P, CDCl_3_) to the alkyl thiophosphinic acids AlkP(S)(OH)H; and in the region of 74–76 ppm to the alkylthiophosphonic acids AlkP(S)(OH)_2_. Further interaction of the above acids with KOH leads to the formation of their potassium salts, which clearly pass into the aqueous layer of the reaction mixture [46,47].

Isolation of the obtained organophosphorus derivatives in individual form from the reaction mixture is a labor-intensive task and presents a separate technological problem. At the same time, the synthesized mixture of alkylphosphine sulfides **4a**–**f** and alkylphosphine oxides **3a**–**f** can be used without separation to study its extraction properties in relation to heavy metals.

**Scheme 2:**
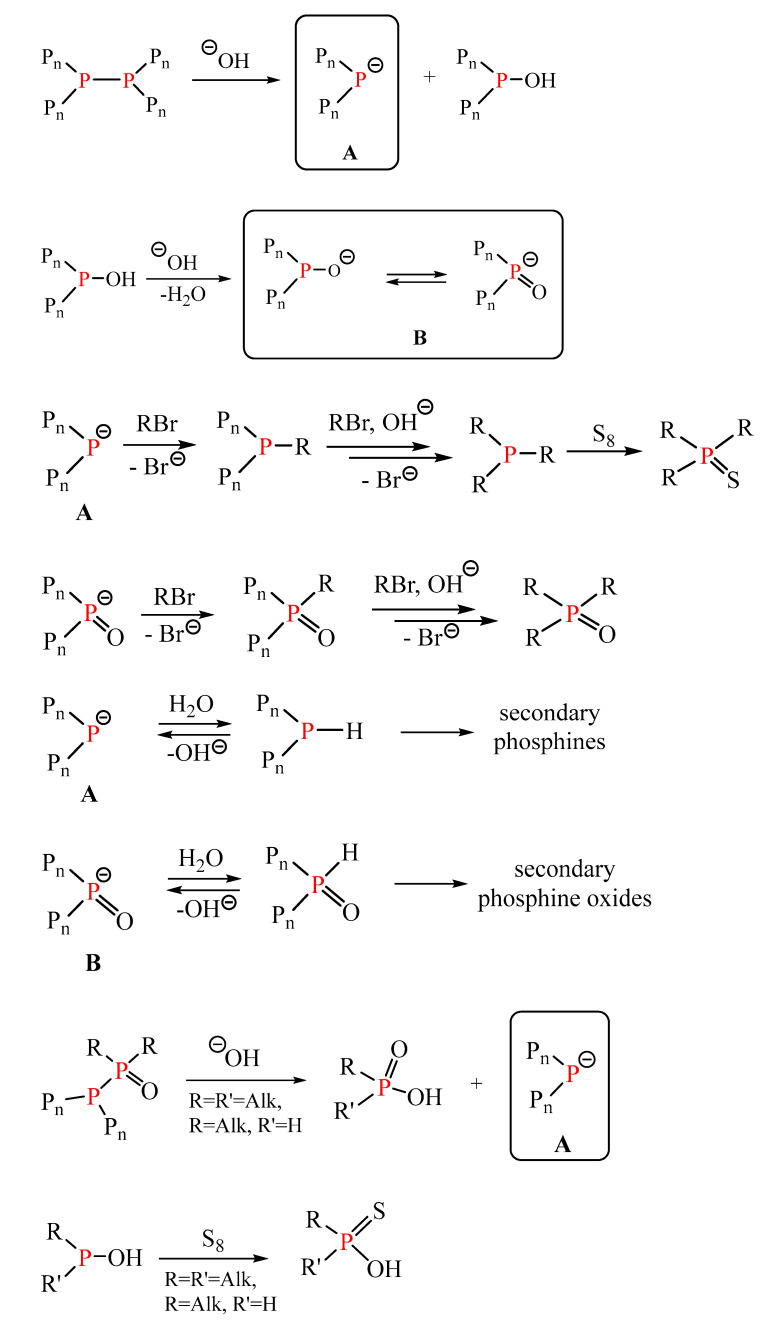
The proposed pathway for the formation of phosphorus-containing products during the reaction of red phosphorus with halogen-containing electrophiles and elemental sulfur in the KOH-toluene-BTEAC system.

The proposed pathway for the formation of phosphorylation reaction products is shown in Scheme 2. The formation of significant amounts of alkylphosphines and alkylphosphine oxides during the phosphorylation of alkyl bromides with red phosphorus in a 60% aqueous solution of KOH-toluene-BTEAC indicates that the process under study proceeds with the participation of both polyphosphide- and polyphosphinite-anions (**A**, **B**). The formation of polyphosphide (**A**) and polyphosphinite (**B**) anions occurs, apparently, as a result of the sequential breaking of P-P bonds in the red phosphorus macromolecule under the action of the OH^−^ anion, which is a sufficiently strong nucleophile and base (Scheme 2) [41].

It can be assumed that the P-nucleophiles formed in situ, polyphosphide- and polyphosphinite-anions (**A**, **B**), are capable of sequentially replacing halogen atoms when interacting with halogen-containing electrophiles and undergoing further cleavage of the P-P bond under the action of hydroxyl groups. This results in the corresponding phosphines and phosphine oxides, as shown in Scheme 2. 

In addition, if the P-P bond breaks faster than the interaction with the electrophile for intermediates **A** and **B**, then the formation of secondary phosphines and phosphine oxides is possible as a result (Scheme 2). Similarly, the implementation of all possible options for breaking P-P bonds in all possible intermediates leads to primary phosphines and phosphine oxides as well as phosphorous-containing acids with one or two alkyl groups. Further, the oxidation of derivatives of trivalent phosphorus with sulfur leads to the corresponding P-, S-containing derivatives.

### 3.2. Extraction Results

Extractions of cobalt(II), lead(II), zinc(II), nickel(II), and silver(I) ions were carried out from acidic solutions (pH = 2–3) using synthesized extractants (mixtures of compounds **3** and **4**) diluted with a solvent. Kerosene, which is widely used in hydrometallurgy, was used as a diluent, thus meeting the requirements of relatively low toxicity, high flash point, and low cost. During the interaction of extractants with solutions, in all cases, the system was stratified into two phases. The boundary of separation of organic and aqueous phases is clear and pronounced; the emulsion or so-called “beard”—the third phase—was not observed.

Studies have shown that a mixture of heptylphosphine oxides and heptylphosphine sulfides (compounds **3a**,**b** and **4a**,**b**) has a high extraction ability in relation to a number of metals. One of the best values of the degree of extraction was shown by the extraction of nickel and cobalt—99.99 and 99.98%. The recovery rate for zinc under the same conditions was 99.95%, and for lead it was 99.90%. Silver is also extracted effectively with this mixture (99.56%).

The elongation of the hydrocarbon chain in the obtained extractants leads to an improvement in the extraction characteristics; this is probably due to a decrease in the electronegativity of the substituent as well as an increase in the hydrophobicity of the extractant. The extraction of nickel and cobalt with a mixture of octylphosphine oxides and octylphosphine sulfides (compounds **3d**,**e** and **4d**,**e**) is close to quantitative (99.99%); this mixture showed a very high extraction ability in relation to zinc and lead: 99.98% and 99.94%, respectively. The process of silver extraction was also highly effective; the degree of extraction was 99.59% (Table 3).

It should be noted that during the extraction of silver using the synthesized extractants (compounds **3** and **4**), the color of the organic phase changed from bright yellow to dark brown. This phenomenon opens up prospects for the use of this extraction mixture in photometric methods for the determination of silver ions in solutions. 

There are characteristic absorption bands of alkyl groups (2853–2953 cm^−1^) in the IR spectra of a mixture of octylphosphine oxides and octylphosphine sulfides taken in a microlayer, as well as signals corresponding to vibrations of fragments P=O (1173–1239 cm^−1^), P-C, and P=S (702–804 cm^−1^) (see Section 2.5.2). As a result of the extraction of silver and lead, there is a significant decrease in the relative intensity of the absorption bands corresponding to the fluctuations of the fragments P=O and P=S (see Section 2.5.3), which apparently indicates the participation of these groups in complex formation.

## 4. Conclusions

When hexyl- and octyl bromides interact with red phosphorus at a temperature of 105–110 °C for 5–6 h (in an argon atmosphere) under the conditions of PTC (BTEAC), mixtures of organophosphorus compounds are formed. Alkylphosphines **2a**–**f** are the main reaction products (57–60%), along with the corresponding alkylphosphine oxides **3a**–**f** (40–43%) which are also formed. The introduction of elemental sulfur at the final stage of the process leads to the expected alkylphosphine sulfides **4a**–**f**, which are the result of the interaction of alkylphosphines present in the reaction medium with sulfur.

It is shown that the synthesized mixtures of phosphorus-containing derivatives can be used in metal extraction processes without separation of components.

The extraction properties of the synthesized mixtures of phosphorus-containing compounds in relation to heavy metals (Ni, Co, Zn, Pb ions) and noble metals (Ag) were tested. It is shown that the obtained mixtures of tertiary phosphine oxides and phosphine sulfides are highly effective extractants; the degree of extraction with respect to Ni, Co, Zn, and Pb varies from 99.90 to 99.99% and for Ag from 99.56 to 99.59%.

Based on the conducted experiments, it can be stated that the target alkylphosphine oxides and alkylphosphine sulfides obtained from readily available starting substances (red phosphorus and technogenic sulfur) are of interest as promising and highly effective extractants and can be widely used in the processes of extraction, concentration, and separation of metals.

## Figures and Tables

**Table 1:** Passport characteristics of sulfur (Tengizchevroil).

Mass Content, %						
**Sulfur**	**Ash**	**Acids in Terms of Sulfuric Acid**	**Organic Substances**	**Arsenic**	**Selenium**	**Water**
99.98	0.0015	0.00045	0.0045	-	-	0.013

**Table 2:** Reaction of red phosphorus with alkyl bromides under PTC, conditions (60% aqueous solution of KOH-toluene-BTEAC) ^a^.

No. Experiment	P_n_, mol (g)	AlkBr, 1a,b mol (g)	S, mol (g)	Temperature, °C	Time, h	Conversion, %		Content in the Mixture, % ^b^	
						Pn	AlkBr	Phosphine Oxides ^c^ 3	Phosphine Sulfides ^c^ 4
1	0.2 (6.2)	C_7_H_15_Br 0.1 (17.01)	0.02 (0.7)	105–110	5	100	69	42 (**3a**,**b**,**c**)	58 (**4a**,**b**,**c**)
2	0.1 (3.1)	C_8_H_17_Br 0.05 (9.66)	-	105–110	6	100	77	40 (**3d**,**e**,**f**)	-
3	0.1 (3.1)	C_8_H_17_Br 0.05 (9.66)	0.01 (0.35)	105–110	6	96	100	43 (**3d**,**e**,**f**)	57 (**4d**,**e**,**f**)
4	0.1 (3.1)	C_8_H_17_Br 0.1 (19.31)	-	90–94	5	80	24	41 (**3d**,**e**,**f**)	-
5	0.1 (3.1)	C_8_H_17_Br 0.15 (28.97)	0.01 (0.35)	105–110	8	61	49	99 (**3d**,**e**,**f**)	-
6	0.1 (3.1)	C_8_H_17_Br 0.175 (33.80)	0.02 (0.7)	100–105	5	85	42	52 (3**d**) ^d^	37 (**4d**)
7	0.1 (3.1)	C_8_H_17_Br 0.03 (5.79)	0.01 (0.35)	100–105	5	100	100	22 (**3d**,**e**,**f**) ^d^	18 (**4d**,**e**,**f**)

Notes. ^a^ Experiment 1: 60 g KOH, 40 mL H_2_O, 1.14 g BTEAC (5 mol%), 90 mL toluene was used. Experiments 2–7: 35 g KOH, 15 mL H_2_O, 0.57 g BTEAC (5 mol%), 45 mL toluene was used. Experiments 2, 4 were carried out without the addition of sulfur, content octylphophines **2d**,**e**,**f**~60%. ^b^ According to NMR ^31^P. ^c^ the total content. ^d^ Additionally experiments were carried out without the addition of sulfur, wherein trioctylphosphine oxide **3d** (experiment 6, 67% yield) and dioctylphosphine oxide **3e** (experiment 6 and 7, 28% yield) were isolated.

**Table 3:** Degree of extraction of metal ions by synthesized extractants.

Extractant	Degree of Extraction E, %				
	Ni(II)	Co(II)	Zn(II)	Pb(II)	Ag(I)
**3a**,**b**, **4a**,**b**	99.99	99.98	99.95	99.90	99.56
**3d**,**e**, **4d**,**e**	99.99	99.99	99.98	99.94	99.59

## Data Availability

Not applicable.

## Supplementary Materials

The following supporting information can be downloaded at: https://www.mdpi.com/article/10.3390/ma16093394/s1. Figures S1 (S1.1–S1.6): IR spectra for compounds **3**, **4** and mixtures; Figures S2 (S2.1–S2.3.7): NMR spectra for compounds **1**–**4**.
