# Supplementary material for: Synthesis and Properties of Sulfur-Containing Organophosphorus Extractants Based on Red Phosphorus, Alkyl Bromides, and Elemental Sulfur
Source: Materials (Basel). 2023 Apr 26;16(9):3394. doi: 10.3390/ma16093394 (PMC10180263; doi:10.3390/ma16093394)

Synthesis and properties of sulfur-containing organophosphorus extractants based on red phosphorus, alkyl bromides, and elemental sulfur

Gaukhar K. Bishimbayeva[1], Nina K. Gusarova[2], Arailym M. Nalibayeva[1*], Svetlana I. Verkhoturova[2], Amangul Bold[1], Natalya A. Chernysheva[2], Assem K. Zhangabayeva[1], Svetlana N. Arbuzova[2], Yerlan N. Abdikalykov[1], Dinara S. Zhumabayeva[1]

[1]D.V. Sokolsky Institute of Fuel, Catalysis and Electrochemistry
050010, Almaty, Kunayev, 142, Kazakhstan
[2]A.E. Favorsky Irkutsk Institute of Chemistry, Siberian Branch Russian Academy of Sciences
664033, Irkutsk, st. Favorskogo, 1, Russia
Correspondence: aray77@mail.ru (A.M.N.)

Keywords: organic phosphine sulfides, organic phosphine oxides, red phosphorus, sulfur, synthesis, metal extractants.

Supplementary Materials

S1. IR data.. 3
S1.1 IR spectrum of a mixture of heptylphosphine oxides **3a,b** and heptylphosphine sulfides **4a,b**.......... 3
S1.2 IR spectrum of a mixture of octylphosphine oxide **3d,e** and octylphosphine sulfides **4d,e**................ 4
S1.3 IR spectra of the organic phase after silver extraction with a mixture of octylphosphine oxide and octylphosphine sulfides in kerosene... 5
S1.4 IR spectra of the organic phase after silver extraction with a mixture of octylphosphine oxide and octylphosphine sulfides in kerosene... 6
S1.5 IR spectra of the organic phase after lead extraction with a mixture of octylphosphine oxide and octylphosphine sulfides in kerosene... 7
S1.6 IR spectra of the organic phase after lead extraction with a mixture of octylphosphine oxide and octylphosphine sulfides in kerosene... 8
S2. NMR data... 9
S2.1 NMR spectra of alkylphosphine oxides **3d,e**.. 9
S2.1.1 ^{1}H NMR spectrum of dioctylphosphine oxide **3e**... 9
S2.1.2 ^{13}C NMR spectrum of dioctylphosphine oxide **3e**.. 10
S2.1.3 $^{31}P_{dec}$ NMR spectrum of dioctylphosphine oxide **3e**.. 11
S2.1.4 $^{31}P_{cop}$ NMR spectrum of dioctylphosphine oxide **3e**.. 12
S2.1.5 ^{1}H NMR spectrum of trioctylphosphine oxide **3d**... 13
S2.1.6 $^{31}P_{dec}$ NMR spectrum of trioctylphosphine oxide **3d**... 14
S2.2 NMR spectra of the reaction of red phosphorus with heptyl bromide **1a** under PTC conditions followed by the introduction of elemental sulfur into the reaction medium... 15
S2.2.1 $^{31}P_{dec}$ NMR spectrum (toluene) of a mixture of heptylphosphines **2a-c** and heptylphosphine oxides **3a-c** prior to the addition of sulfur.. 15
S2.2.2 $^{31}P_{cop}$ NMR spectrum (toluene) of a mixture of heptylphosphines **2a-c** and heptylphosphine oxides **3a-c** prior to the addition of sulfur.. 16
S2.2.3 ^{1}H NMR spectrum ($CDCl_3$) of a mixture of heptylphosphine oxides **3a-c** and heptylphosphine sulfides **4a-c**.. 17
S2.2.4 $^{31}P_{dec}$ NMR spectrum ($CDCl_3$) of a mixture of heptylphosphine oxides **3a-c** and heptylphosphine sulfides **4a-c**.. 18
S2.2.5 $^{31}P_{cop}$ NMR spectrum ($CDCl_3$) of a mixture of heptylphosphine oxides **3a-c** and heptylphosphine sulfides **4a-c**.. 19
S2.3 NMR spectra of the reaction of red phosphorus with octyl bromide **1b** under PTC conditions followed by the introduction of elemental sulfur into the reaction medium...................................... 20
S2.3.1 $^{31}P_{dec}$ NMR spectrum (toluene) of a mixture of octylphosphines **2d-f** and octylphosphine oxides **3d-f** before the addition of sulfur... 20
S2.3.2 $^{31}P_{cor}$ (toluene) NMR spectrum of a mixture of octylphosphines **2d-f** and octylphosphine oxides **3d-f** before the addition of sulfur... 21
S2.3.3 ^{1}H NMR spectrum ($CDCl_3$) of a mixture of octylphosphine oxides **3d-f** and octylphosphine sulfides **4d-f**.. 22
S2.3.4 $^{31}P_{dec}$ NMR spectrum ($CDCl_3$) of a mixture of octylphosphine oxides **3d-f** and octylphosphine sulfides **4d-f**.. 23
S2.3.5 $^{31}P_{cop}$ NMR spectrum ($CDCl_3$) of a mixture of octylphosphine oxides **3d-f** and octylphosphine sulfides **4d-f**.. 24
S2.3.6 $^{31}P_{dec}$ NMR spectrum of a mixture of dioctylphosphine and trioctylphosphine oxides **3d,e**............ 25
S2.3.7 $^{31}P_{cop}$ NMR spectrum of a mixture of dioctylphosphine and trioctylphosphine oxides **3d,e**............ 26

S1. IR data

S1.1 IR spectrum of a mixture of heptylphosphine oxides **3a,b** and heptylphosphine sulfides **4a,b.**

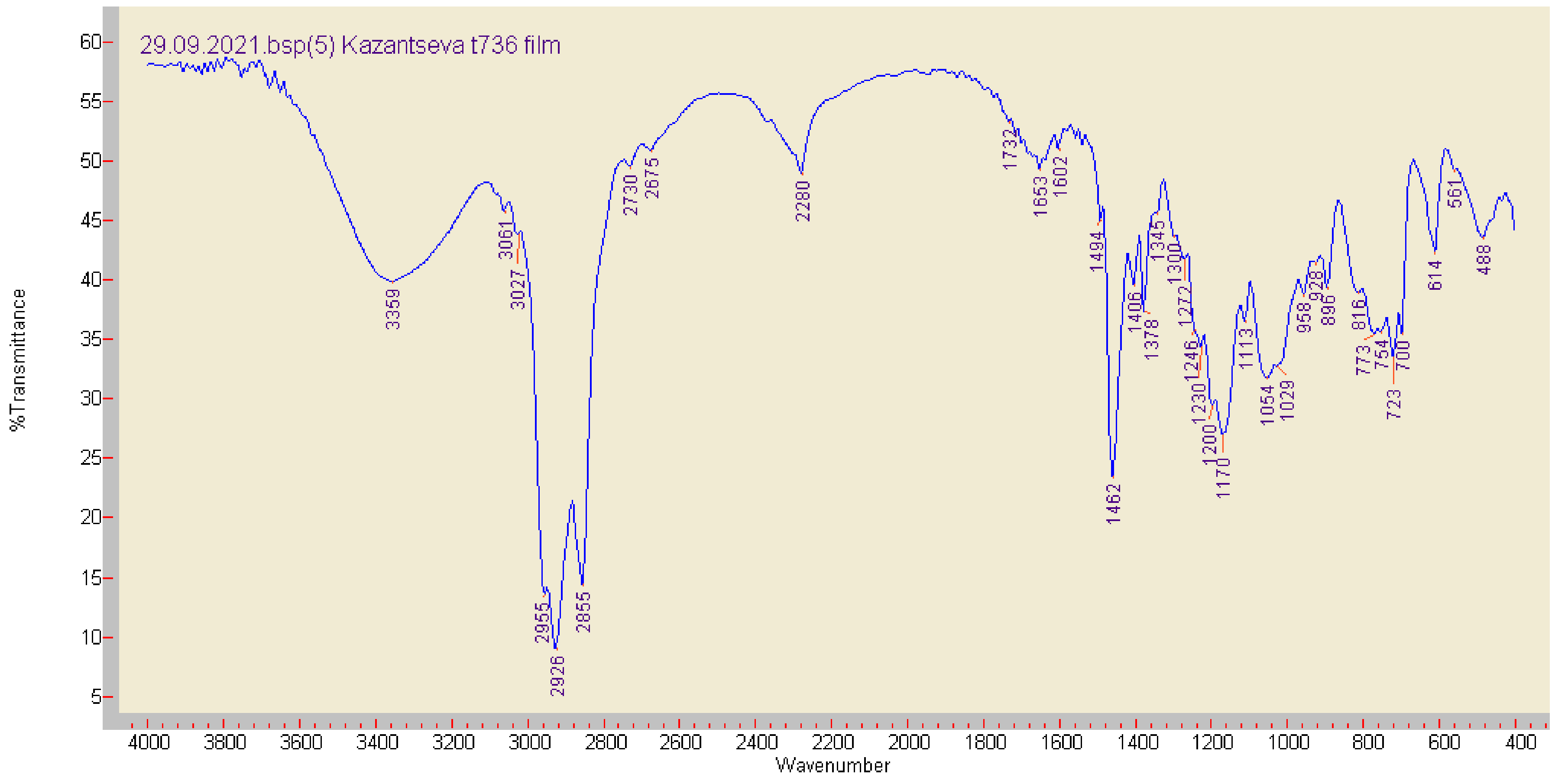

S1.2 IR spectrum of a mixture of octyl phosphine oxide **3d,e** and octyl phosphine sulfides **4d,e.**

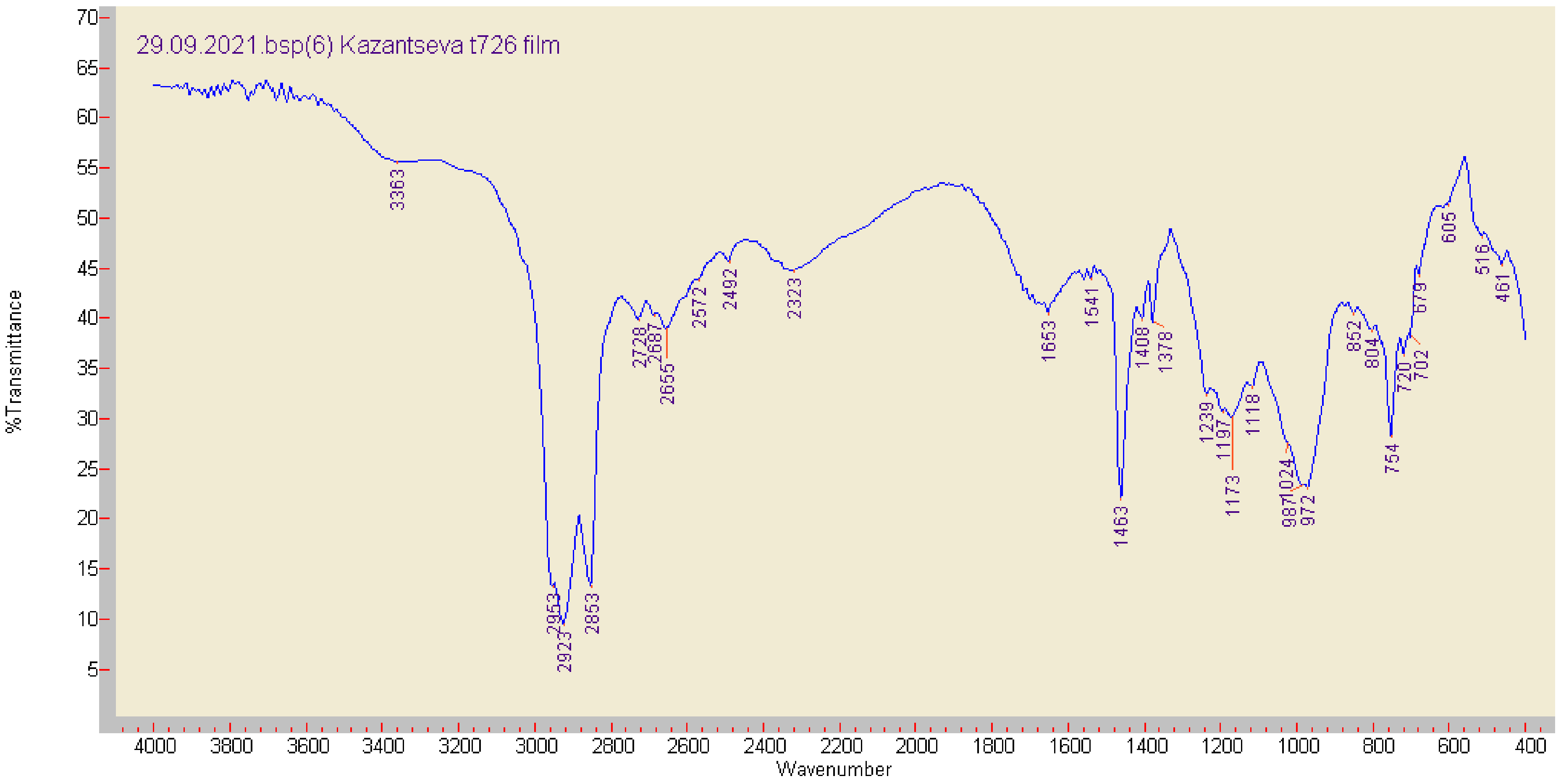

S1.3 IR spectra of the organic phase after silver extraction with a mixture of octylphosphine oxide and octylphosphine sulfides in kerosene (on the y-axis, transmission; on the abscissa, wavenumber (cm^{-1})).

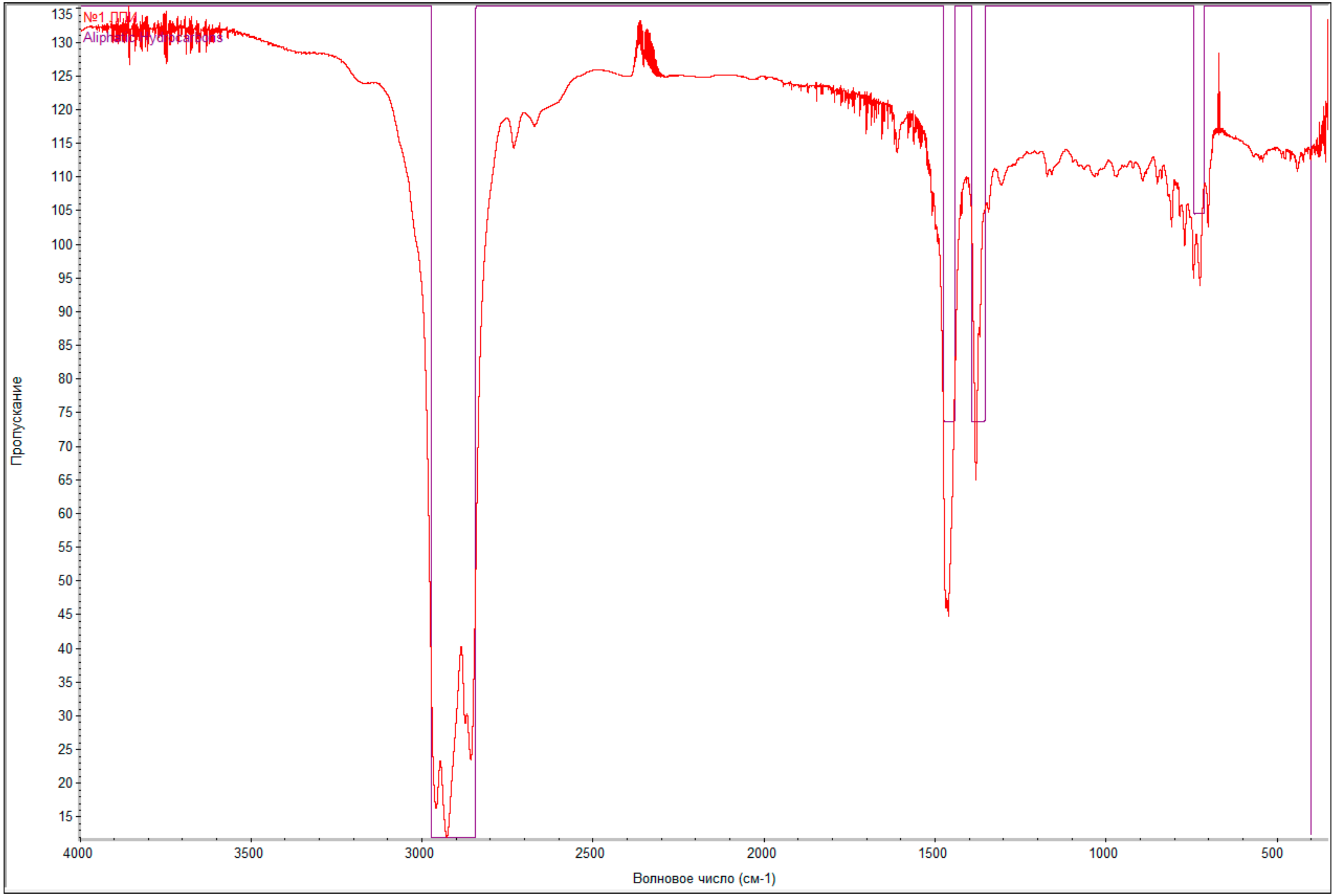

S1.4 IR spectra of the organic phase after silver extraction with a mixture of octylphosphine oxide and octylphosphine sulfides in kerosene (on the y-axis, absorbance; on the abscissa, wavenumber (cm^{-1})).

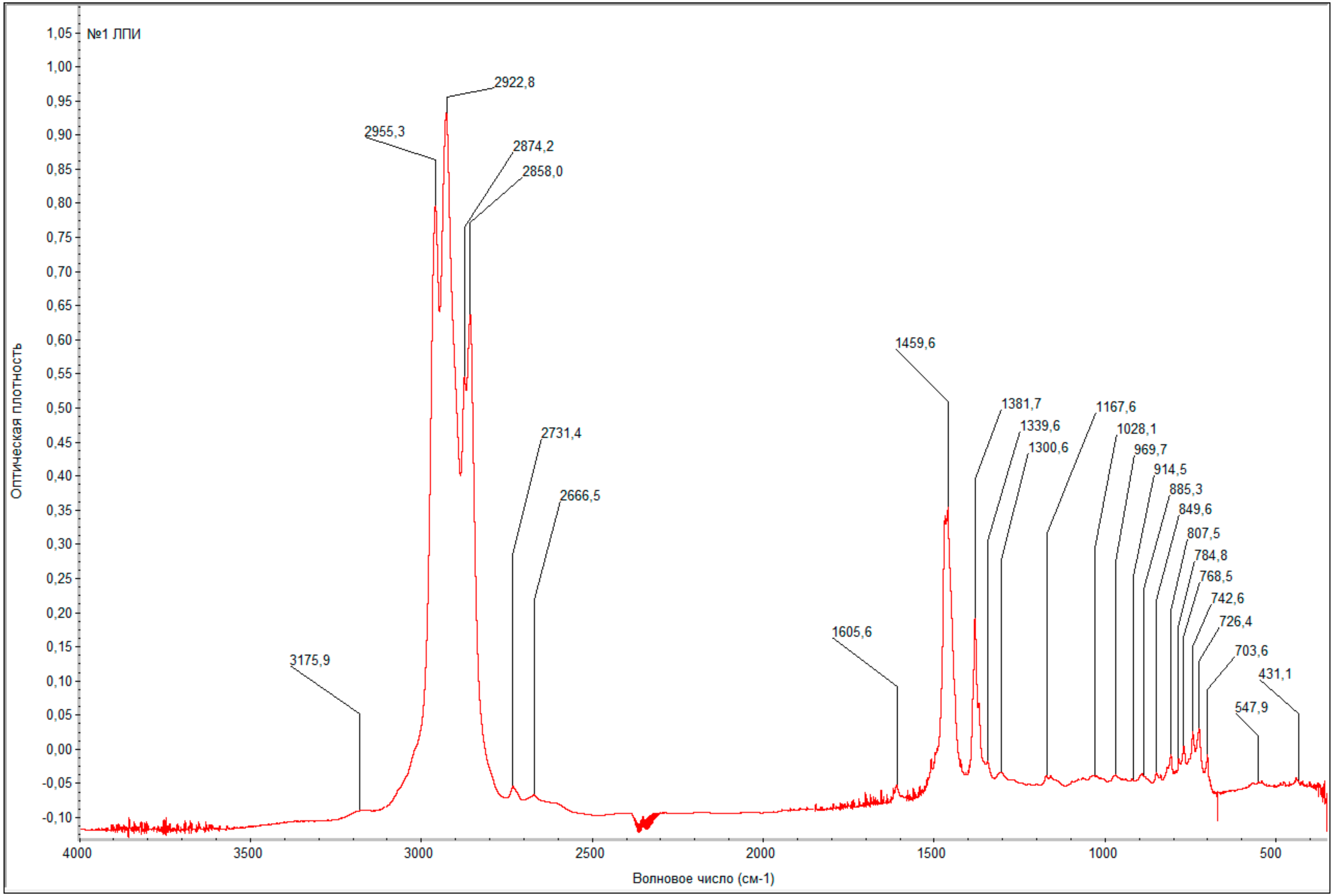

S1.5 IR spectra of the organic phase after lead extraction with a mixture of octylphosphine oxide and octylphosphine sulfides in kerosene (on the y-axis, transmission; on the abscissa, wavenumber (cm^{-1})).

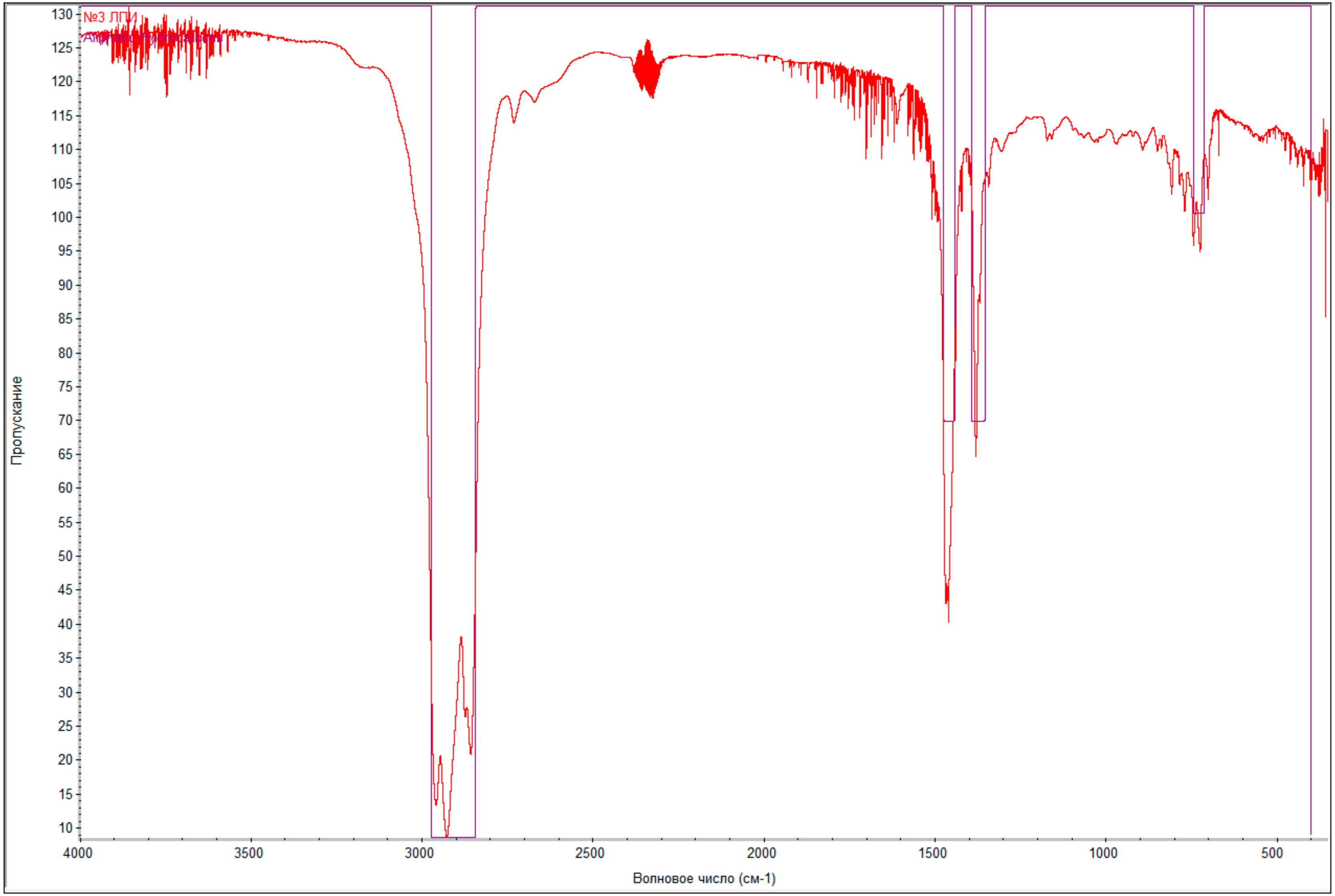

S1.6 IR spectra of the organic phase after lead extraction with a mixture of octylphosphine oxide and octylphosphine sulfides in kerosene (on the y-axis, absorbance; on the abscissa, wavenumber (cm^{-1})).

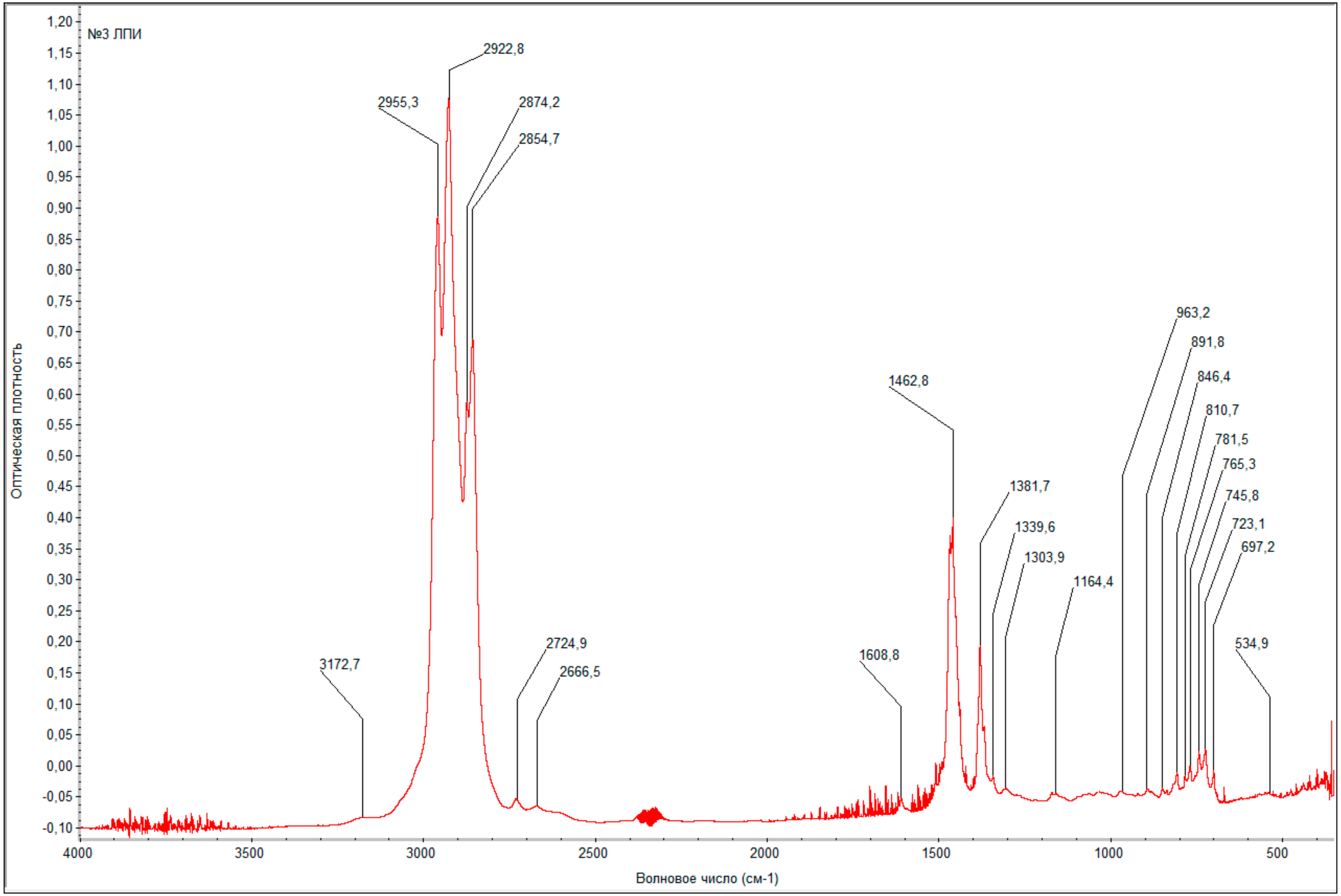

S2. NMR data

S2.1 NMR spectra of alkylphosphine oxides

S2.1.1 ^{1}H NMR spectrum (CDCl3) of dioctylphosphine oxide **3e** (Table 2, experiment No. 7).

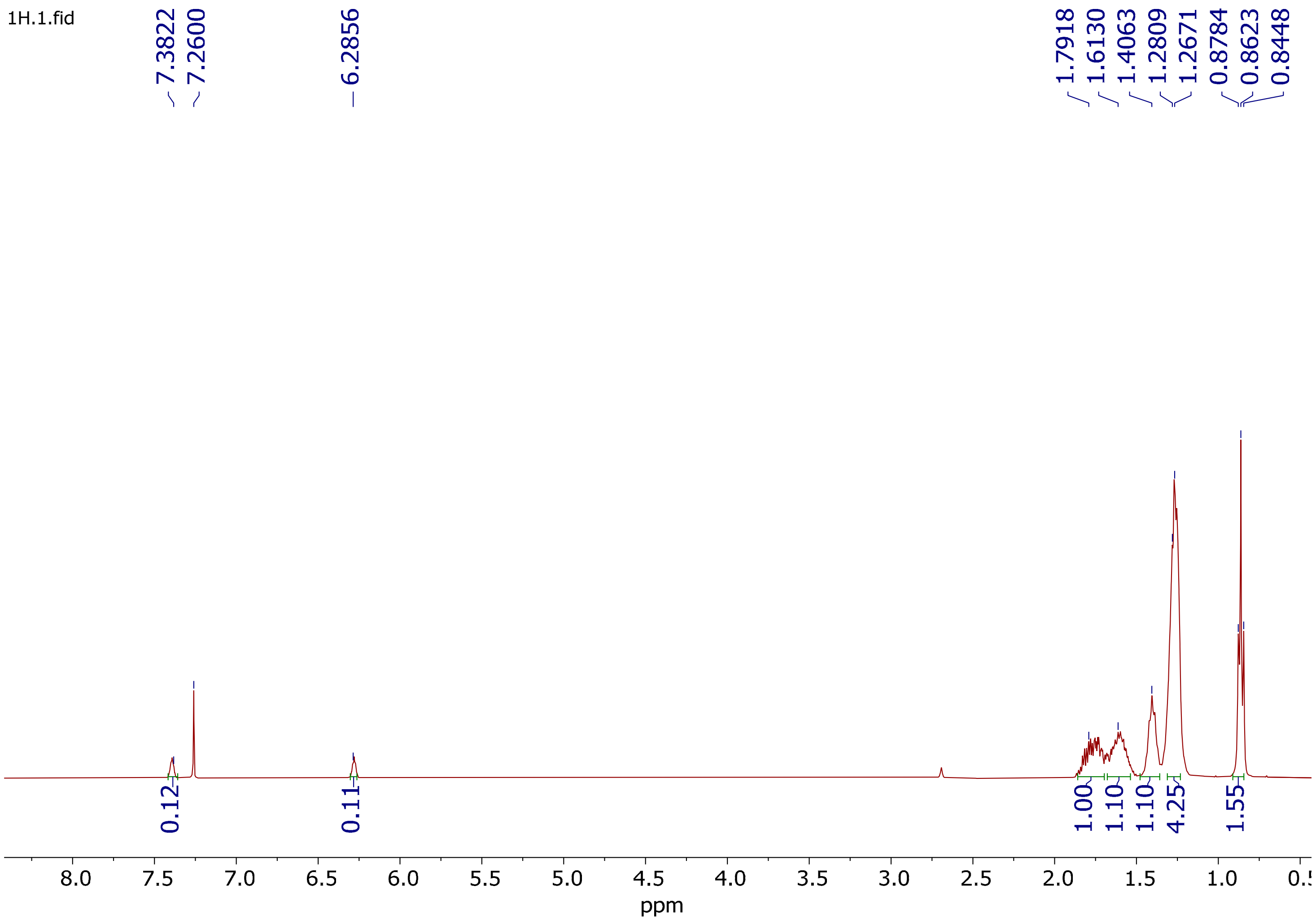

S2.1.2 ^{13}C NMR spectrum ($CDCl_3$) of dioctylphosphine oxide **3e** (Table 2, experiment No. 7).

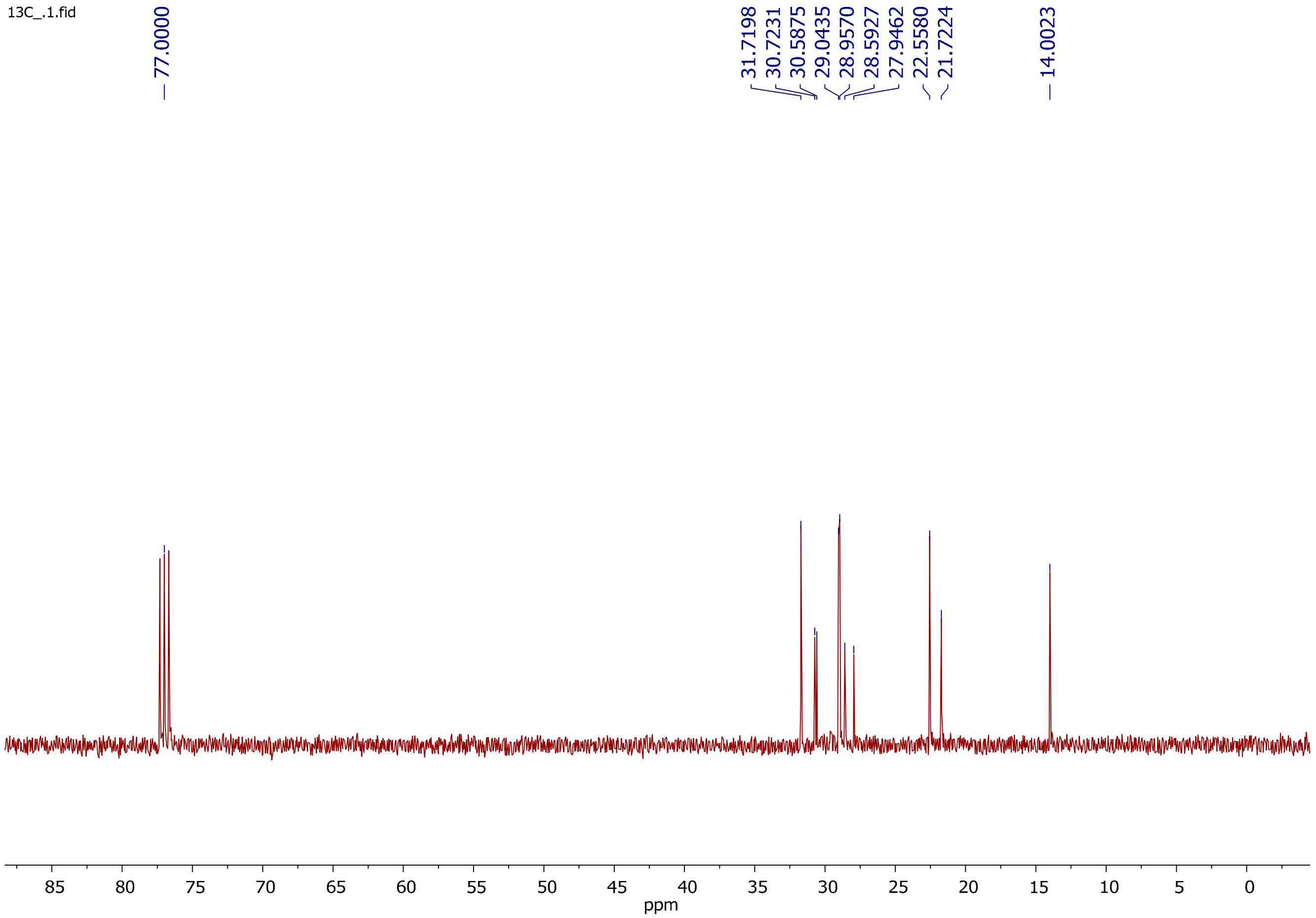

S2.1.3 $^{31}P_{dec}$ NMR spectrum ($CDCl_3$) of dioctylphosphine oxide **3e** (Table 2, experiment No. 7).

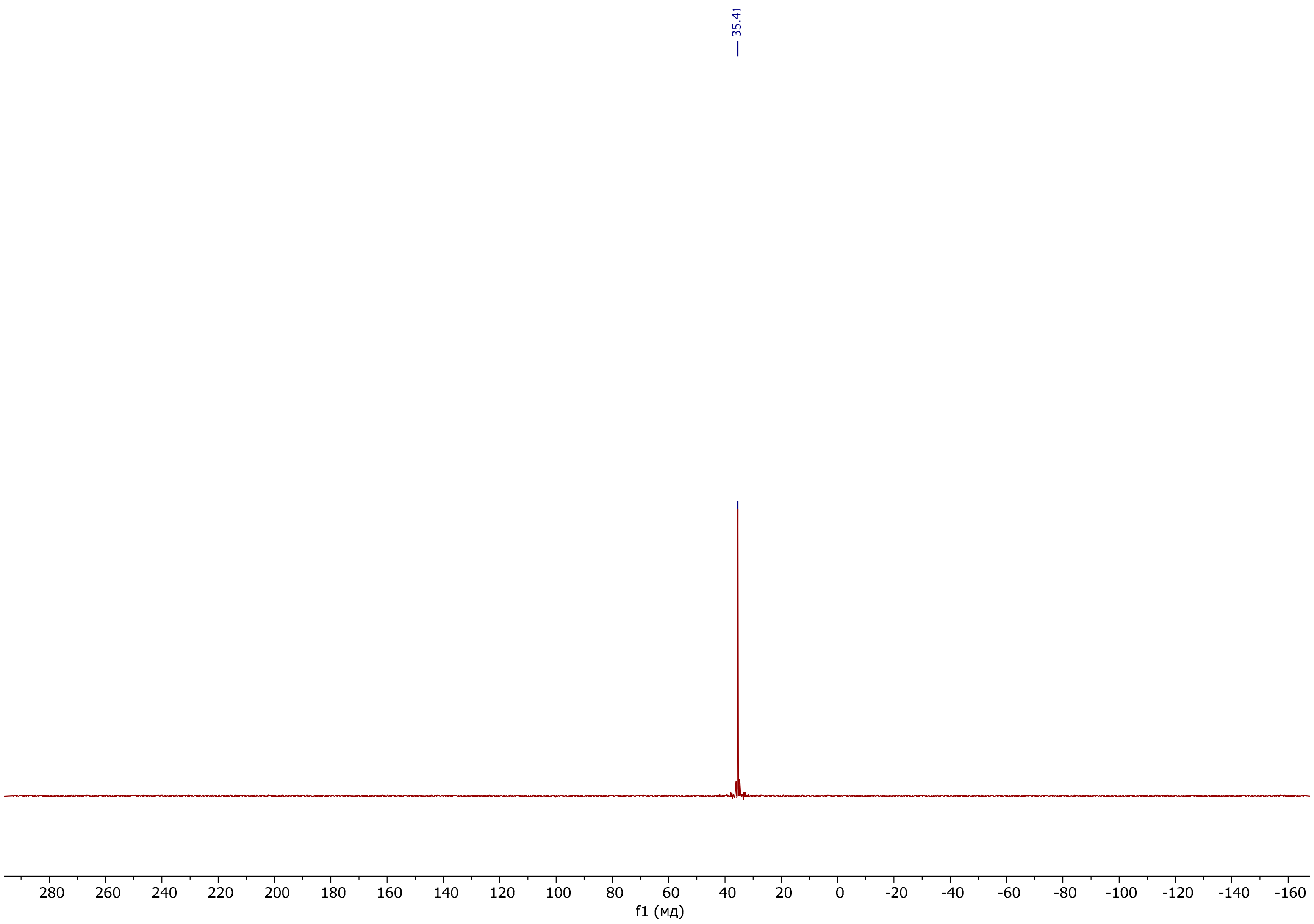

S2.1.4 $^{31}P_{cop}$ NMR spectrum ($CDCl_3$) of dioctylphosphine oxide **3e** (Table 2, experiment No. 7).

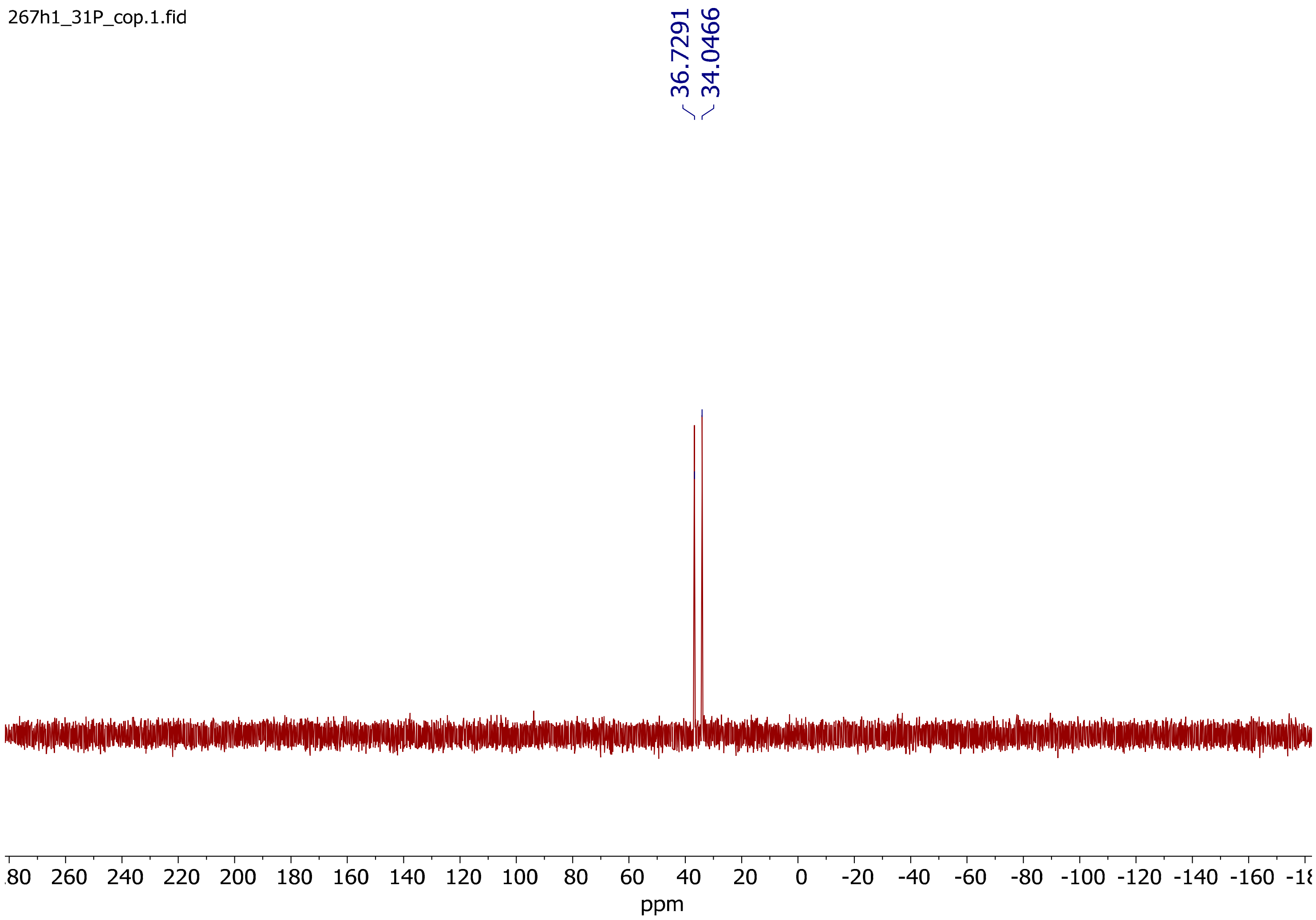

S2.1.5 ^{1}H NMR spectrum ($CDCl_3$) of trioctylphosphine oxide **3d** (Table 2, experiment No. 6).

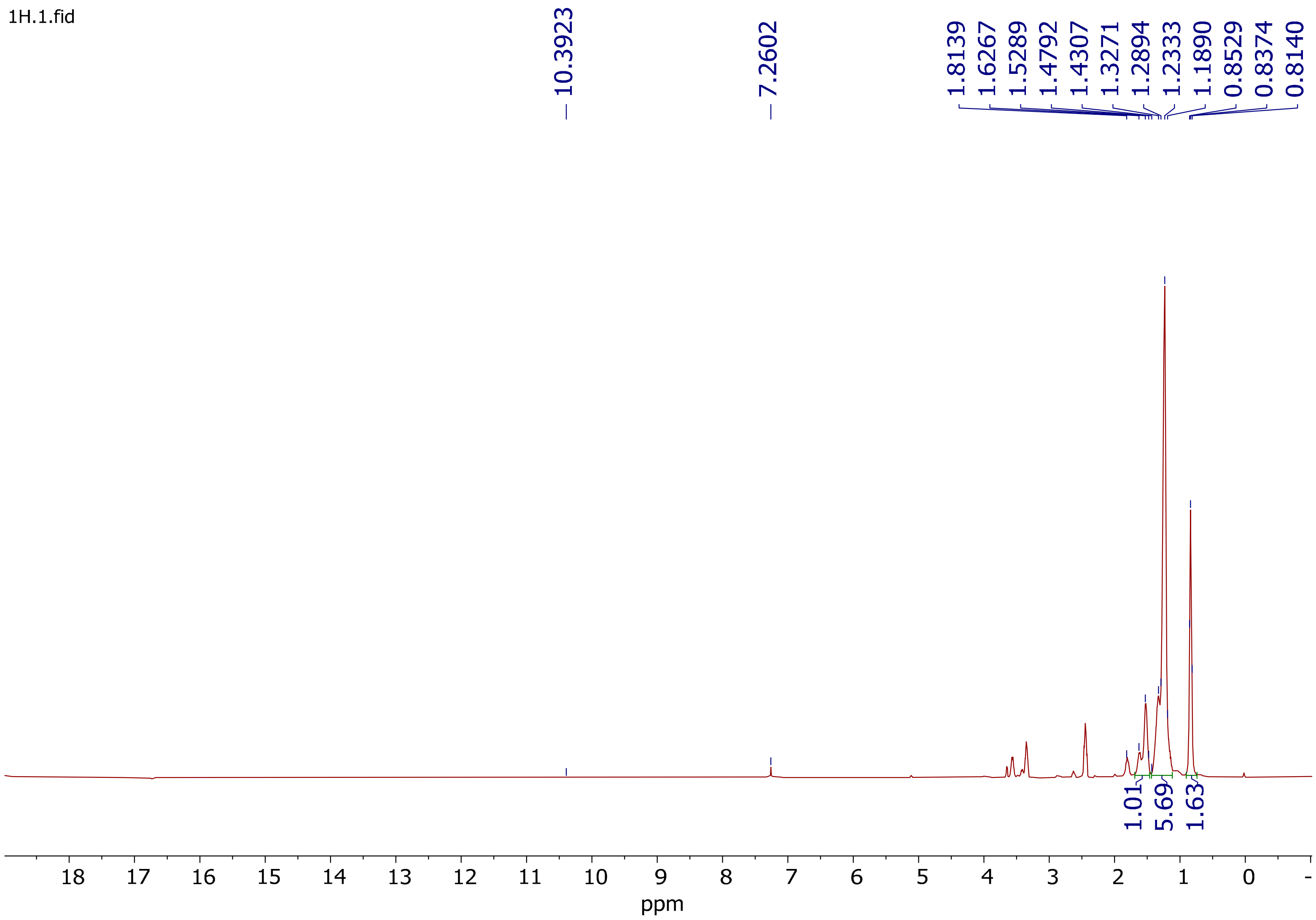

S2.1.6 $^{31}P_{dec}$ NMR spectrum ($CDCl_3$) of trioctylphosphine oxide **3d** (Table 2, experiment No. 6).

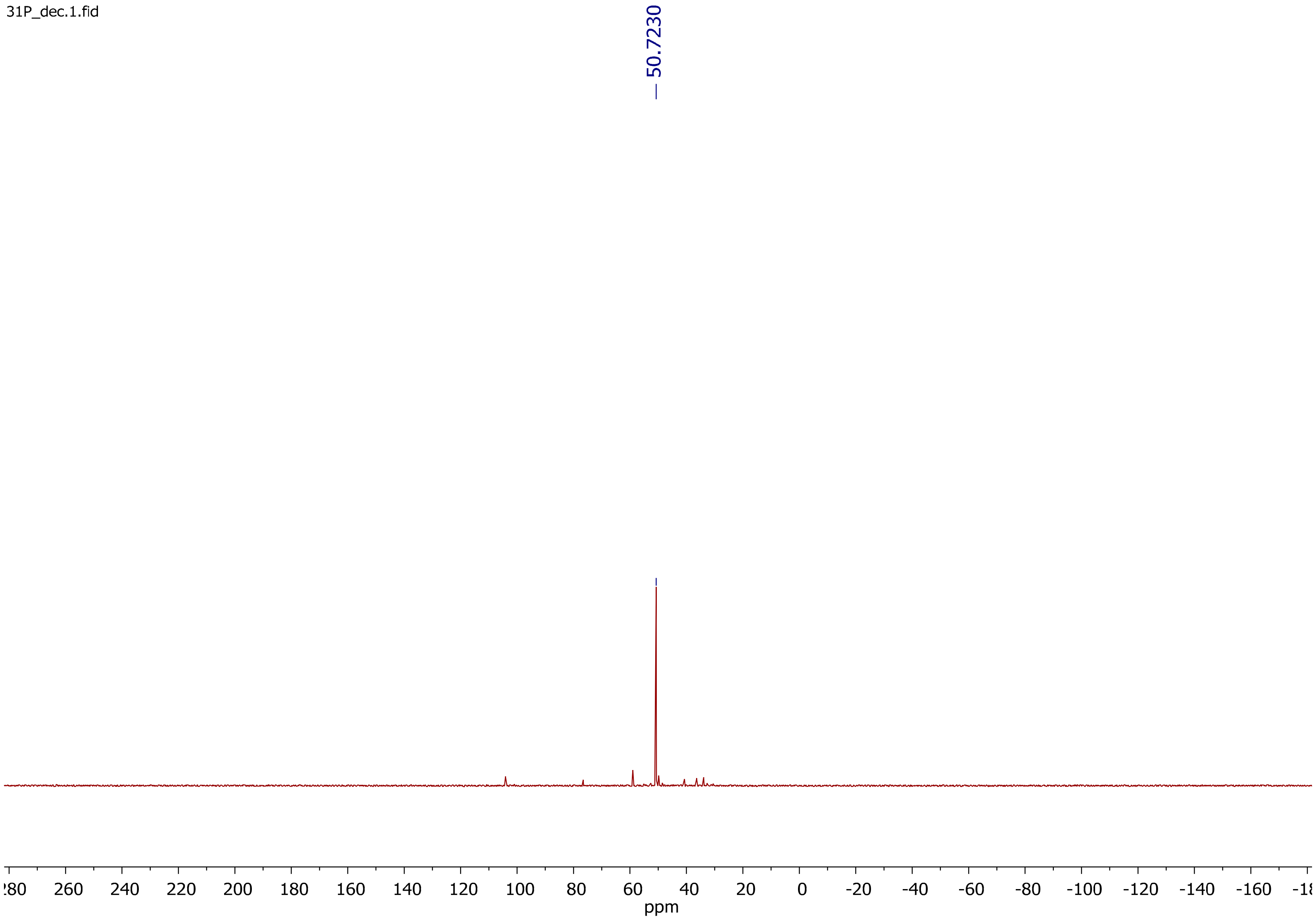

S2.2 NMR spectra of the reaction of red phosphorus with heptyl bromide **1a** under PTC conditions followed by the introduction of elemental sulfur into the reaction medium.

S2.2.1 $^{31}P_{dec}$ NMR spectrum (toluene) of a mixture of heptylphosphines **2a-c** and heptylphosphine oxides **3a-c** prior to the addition of sulfur (Table 2, experiment No. 1).

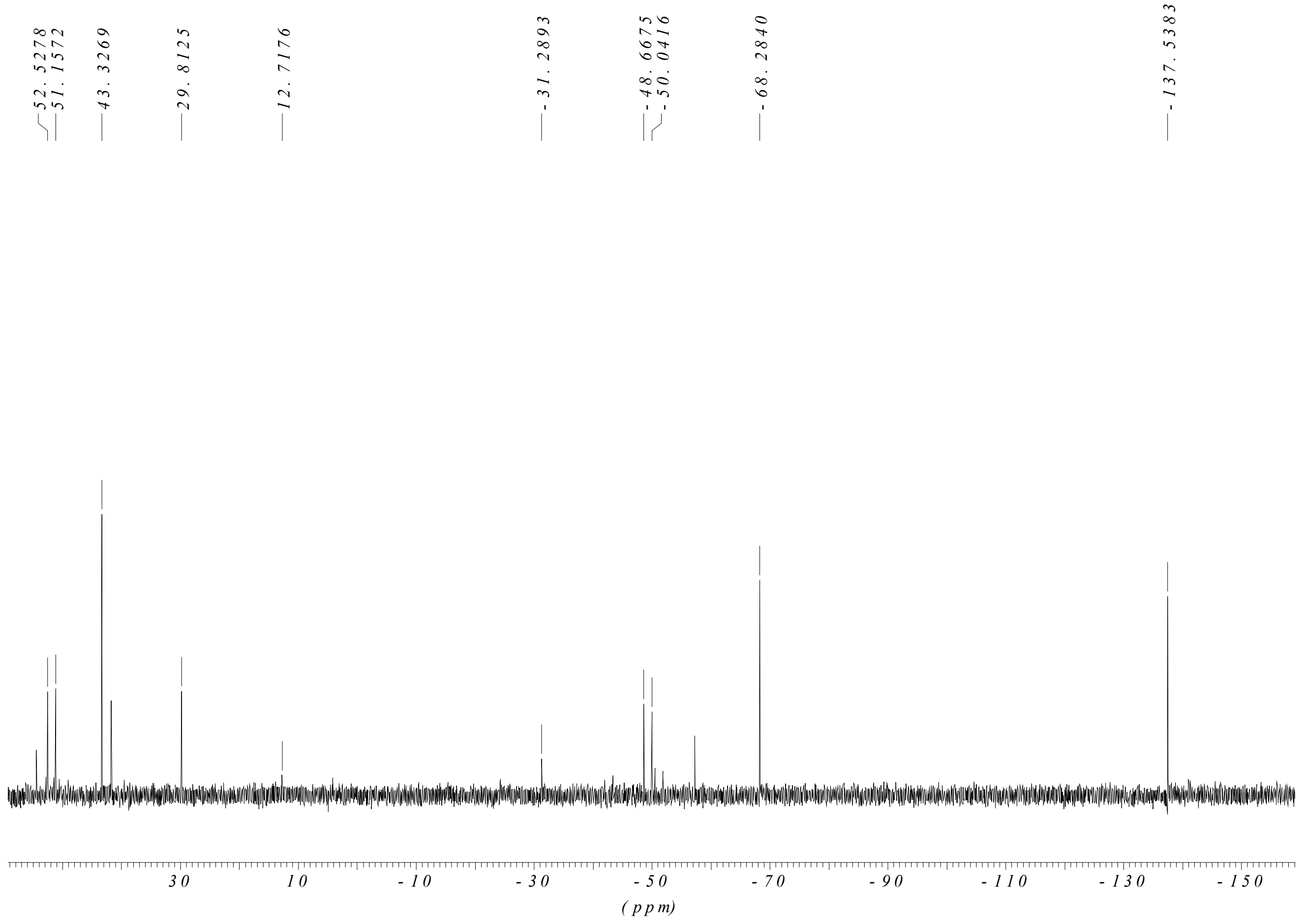

S2.2.2 $^{31}P_{cop}$ NMR spectrum (toluene) of a mixture of heptylphosphines **2a-c** and heptylphosphine oxides **3a-c** prior to the addition of sulfur (Table 2, experiment No. 1).

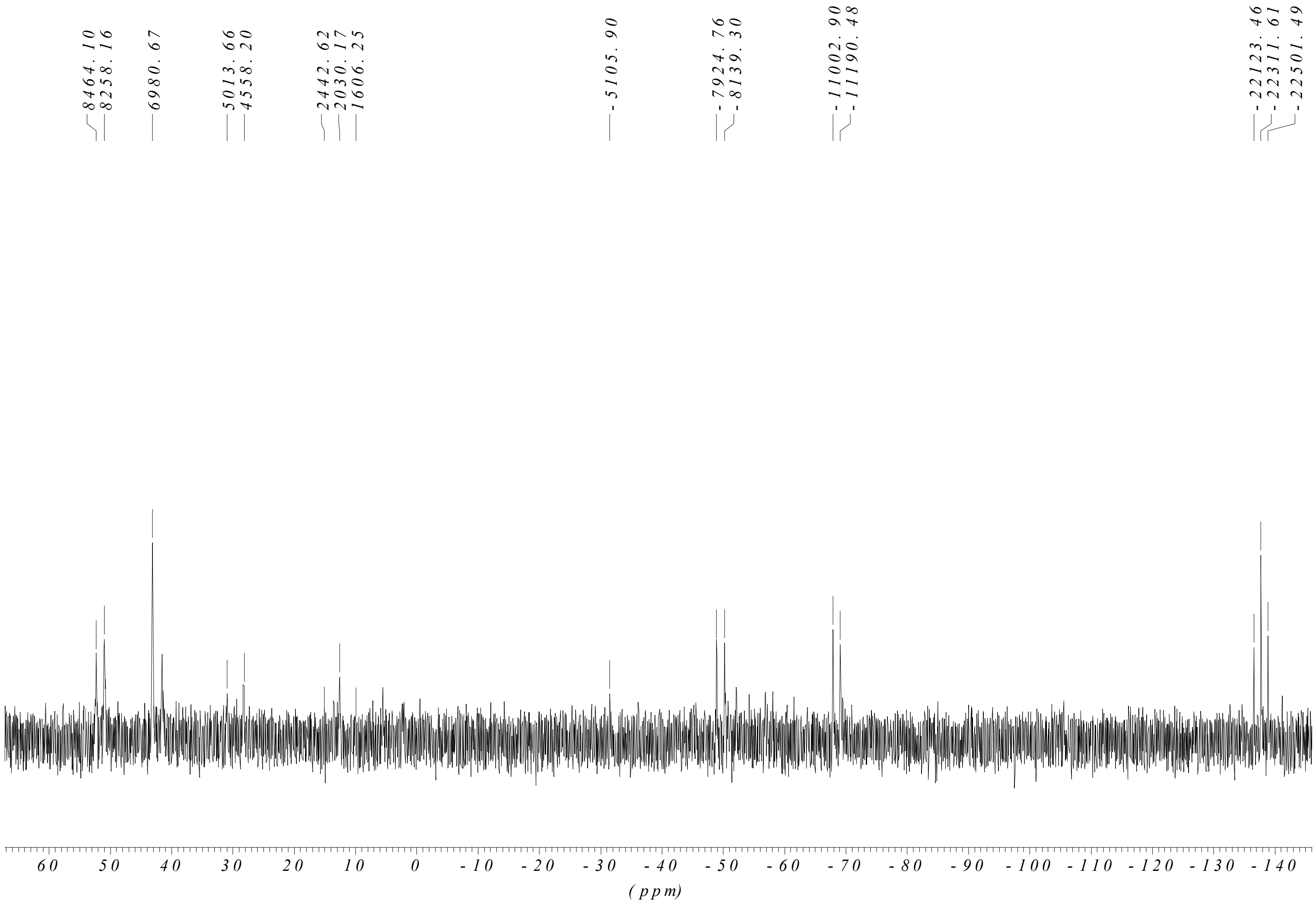

S2.2.3 ^{1}H NMR spectrum ($CDCl_3$) of a mixture of heptylphosphine oxides **2a-c** and heptylphosphine sulfides **3a-c** (Table 2, experiment No. 1).

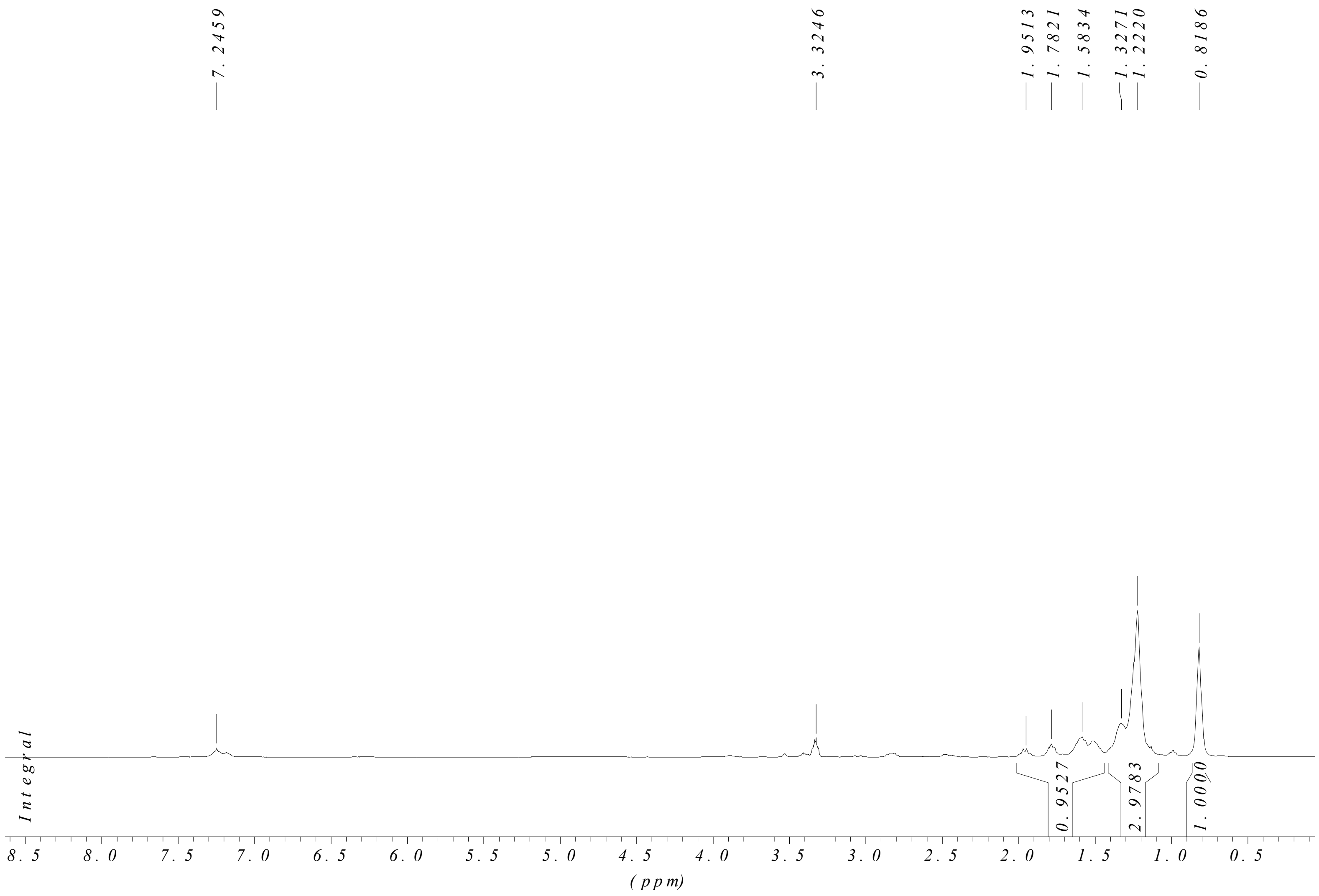

S2.2.4 $^{31}P_{dec}$ NMR spectrum ($CDCl_3$) of a mixture of heptylphosphine oxides **3a-c** and heptylphosphine sulfides **4a-c** (Table 2, experiment No. 1).

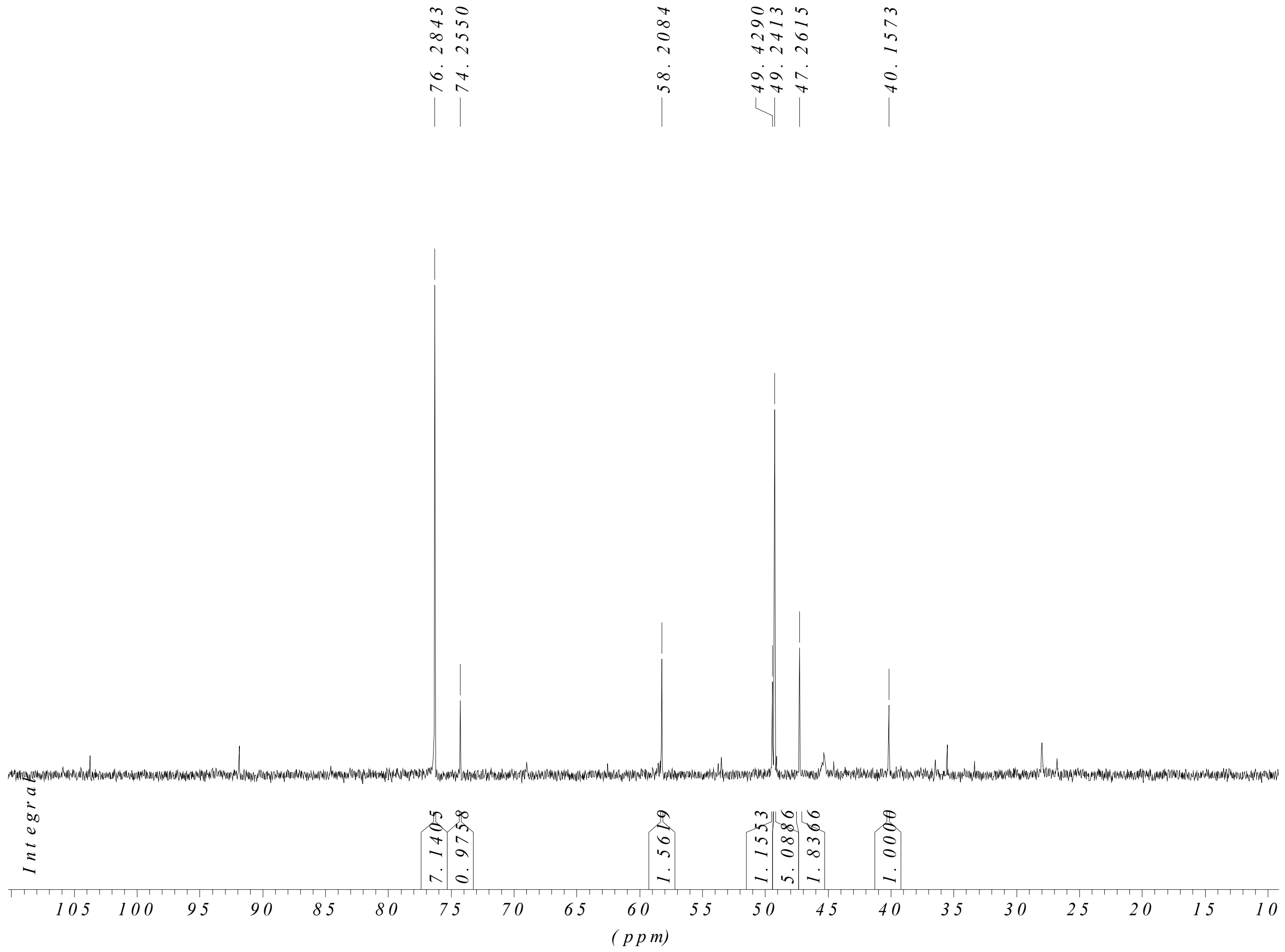

S2.2.5 $^{31}P_{cop}$ NMR spectrum ($CDCl_3$) of a mixture of heptylphosphine oxides **3a-c** and heptylphosphine sulfides **4a-c** (Table 2, experiment No. 1).

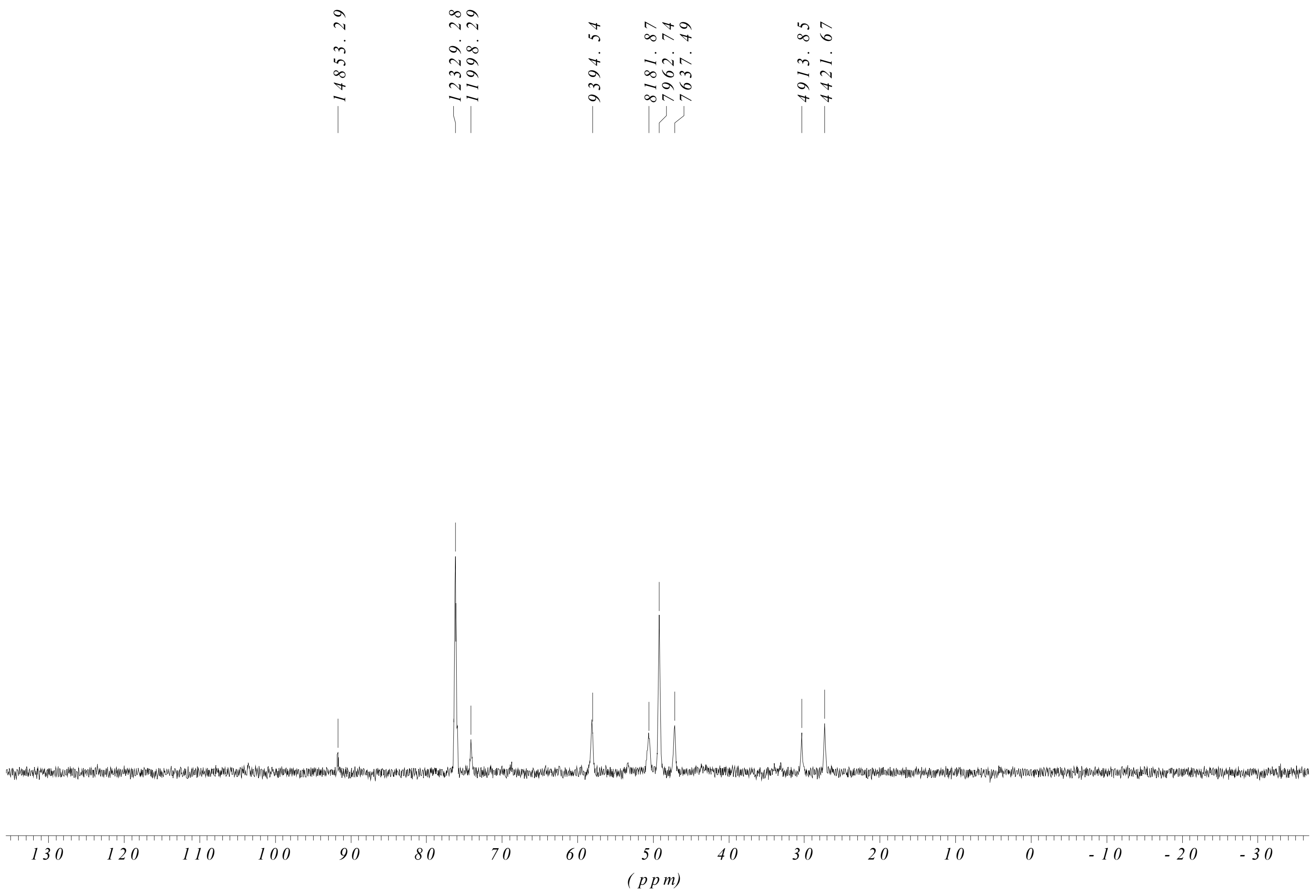

S2.3 NMR spectra of the reaction of red phosphorus with octyl bromide **1b** under PTC conditions followed by the introduction of elemental sulfur into the reaction medium.

S2.3.1 $^{31}P_{dec}$ NMR spectrum (toluene) of a mixture of octylphosphines **2d-f** and octylphosphine oxides **3d-f** before the addition of sulfur (Table 2, experiment No. 3).

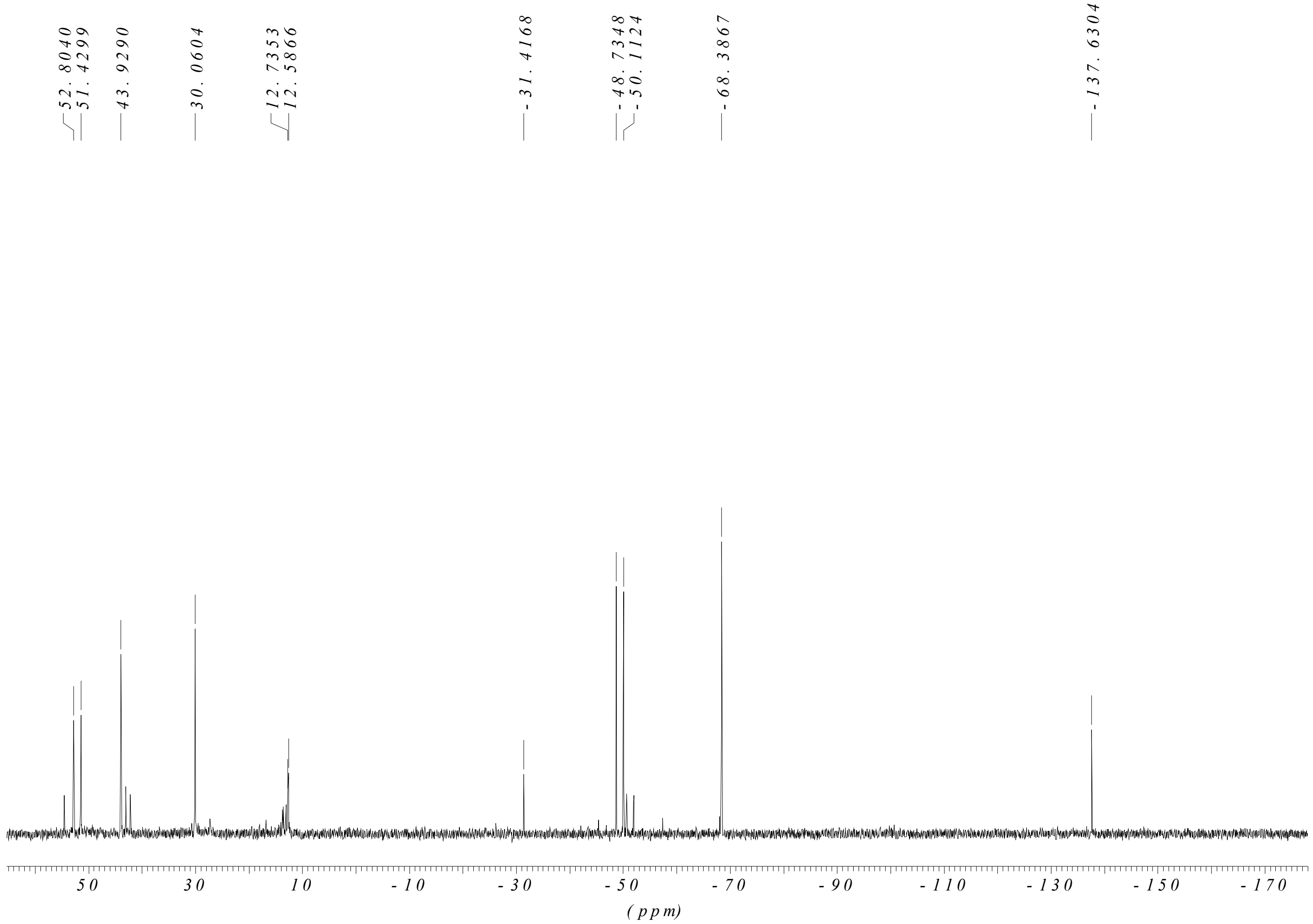

S2.3.2 $^{31}P_{cop}$ (toluene) NMR spectrum of a mixture of octylphosphines **2d-f** and octylphosphine oxides **3d-f** before the addition of sulfur (Table 2, experiment No. 3).

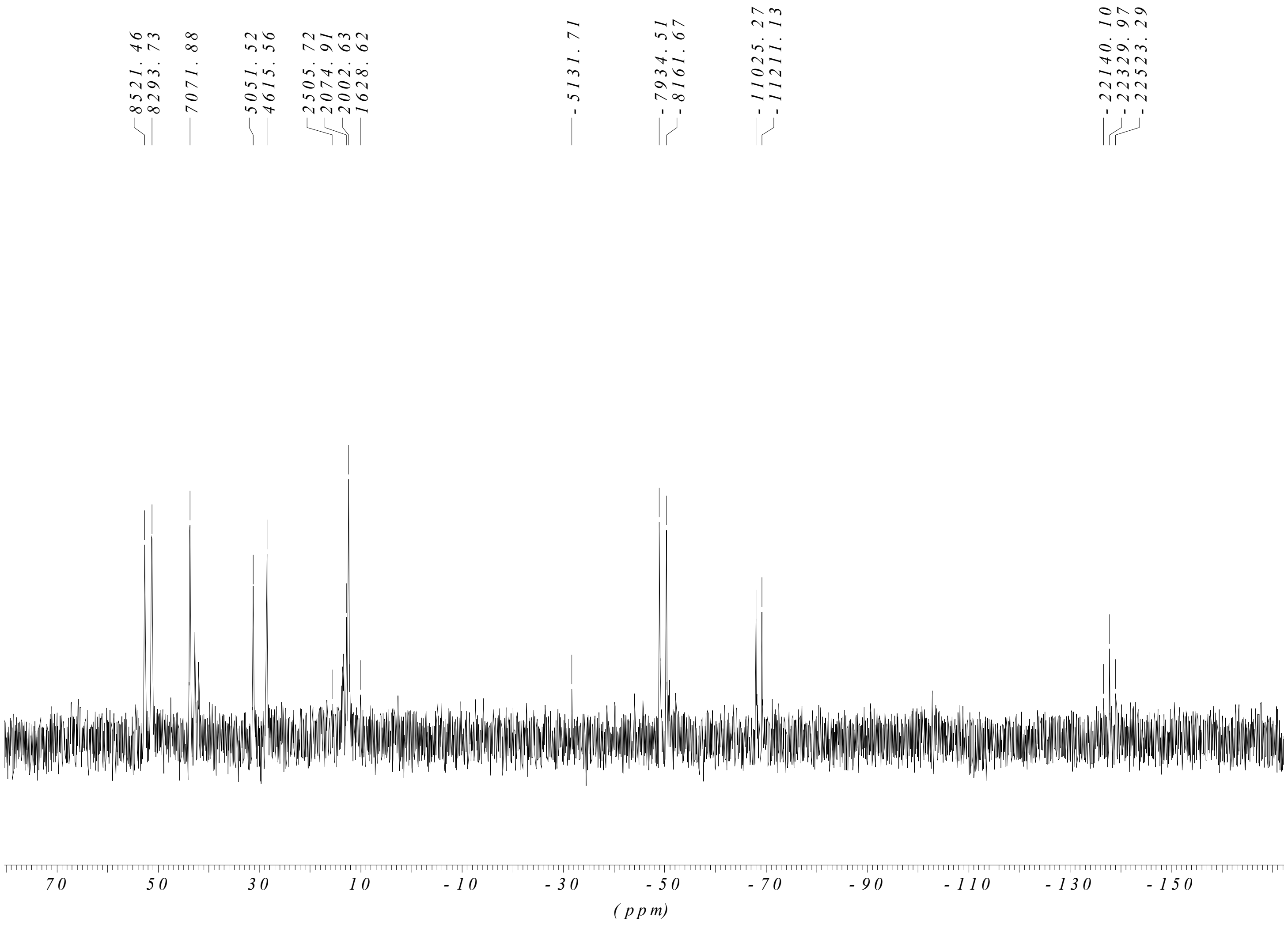

S2.3.3 ^{1}H NMR spectrum ($CDCl_3$) of a mixture of octylphosphine oxides **3d-f** and octylphosphine sulfides **4d-f** (Table 2, experiment No. 3).

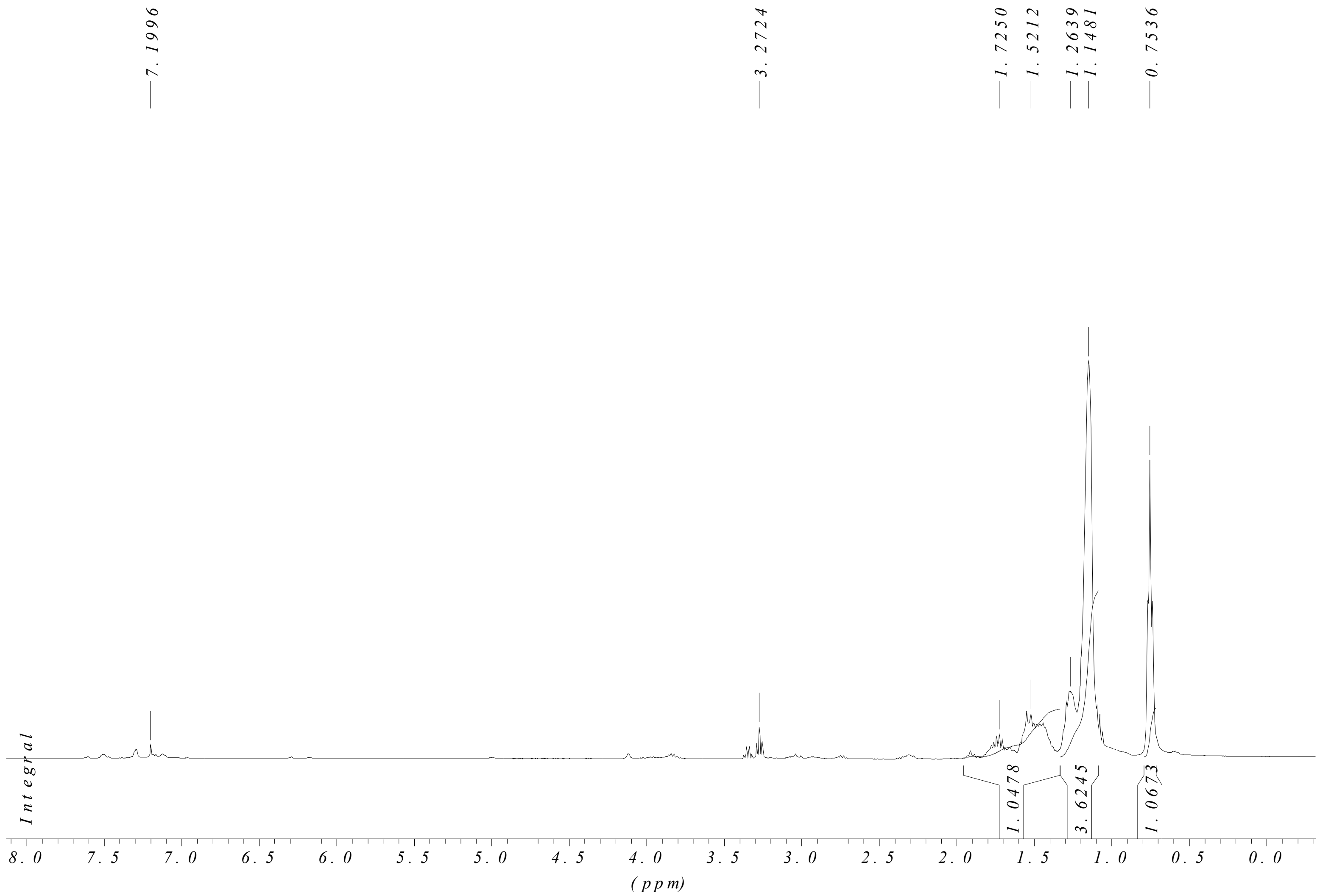

S2.3.4 $^{31}P_{dec}$ NMR spectrum ($CDCl_3$) of a mixture of octylphosphine oxides **3d-f** and octylphosphine sulfides **4d-f** (Table 2, experiment No. 3).

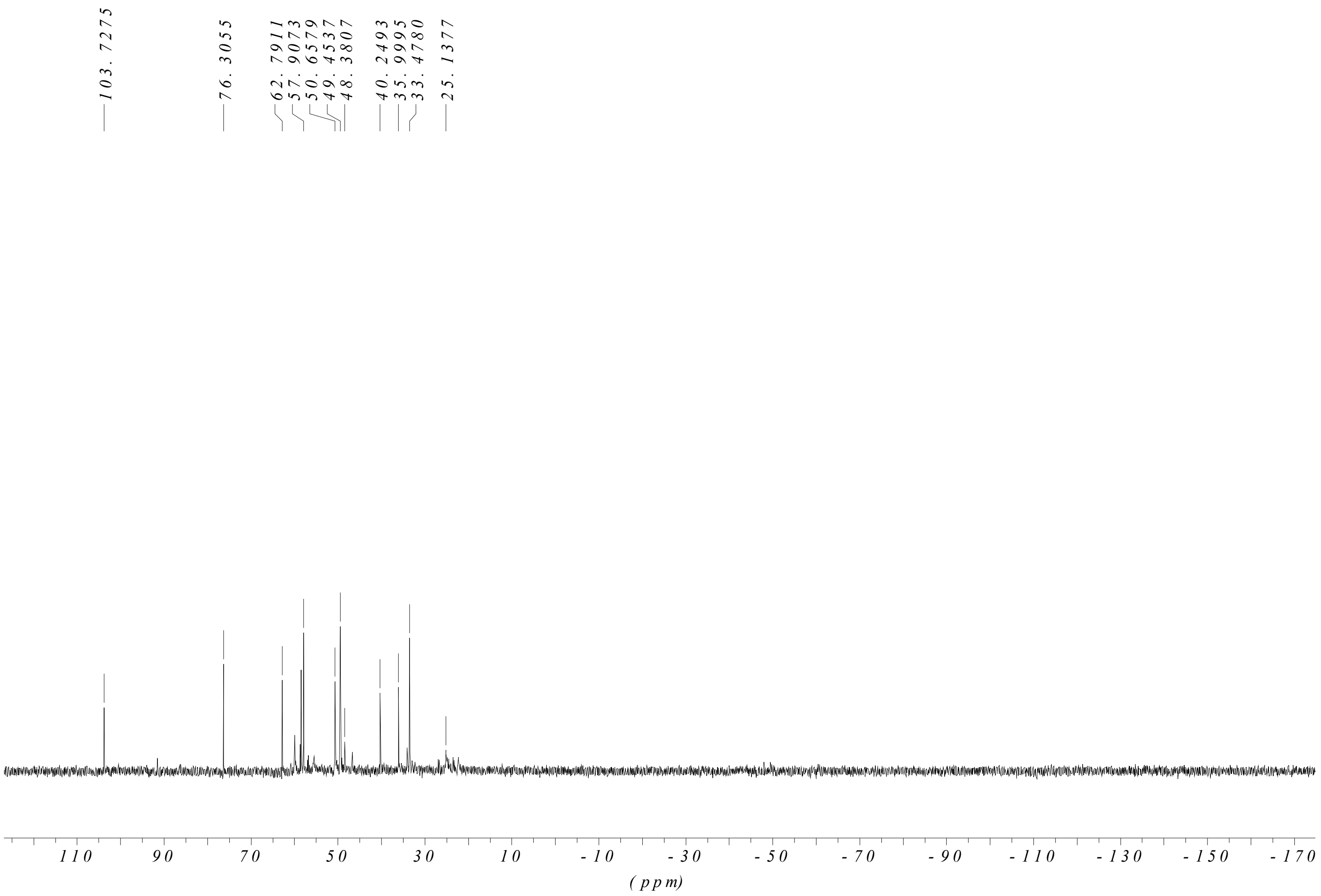

S2.3.5 $^{31}P_{cop}$ NMR spectrum ($CDCl_3$) of a mixture of octylphosphine oxides **3d-f** and octylphosphine sulfides **4d-f** (Table 2, experiment No. 3).

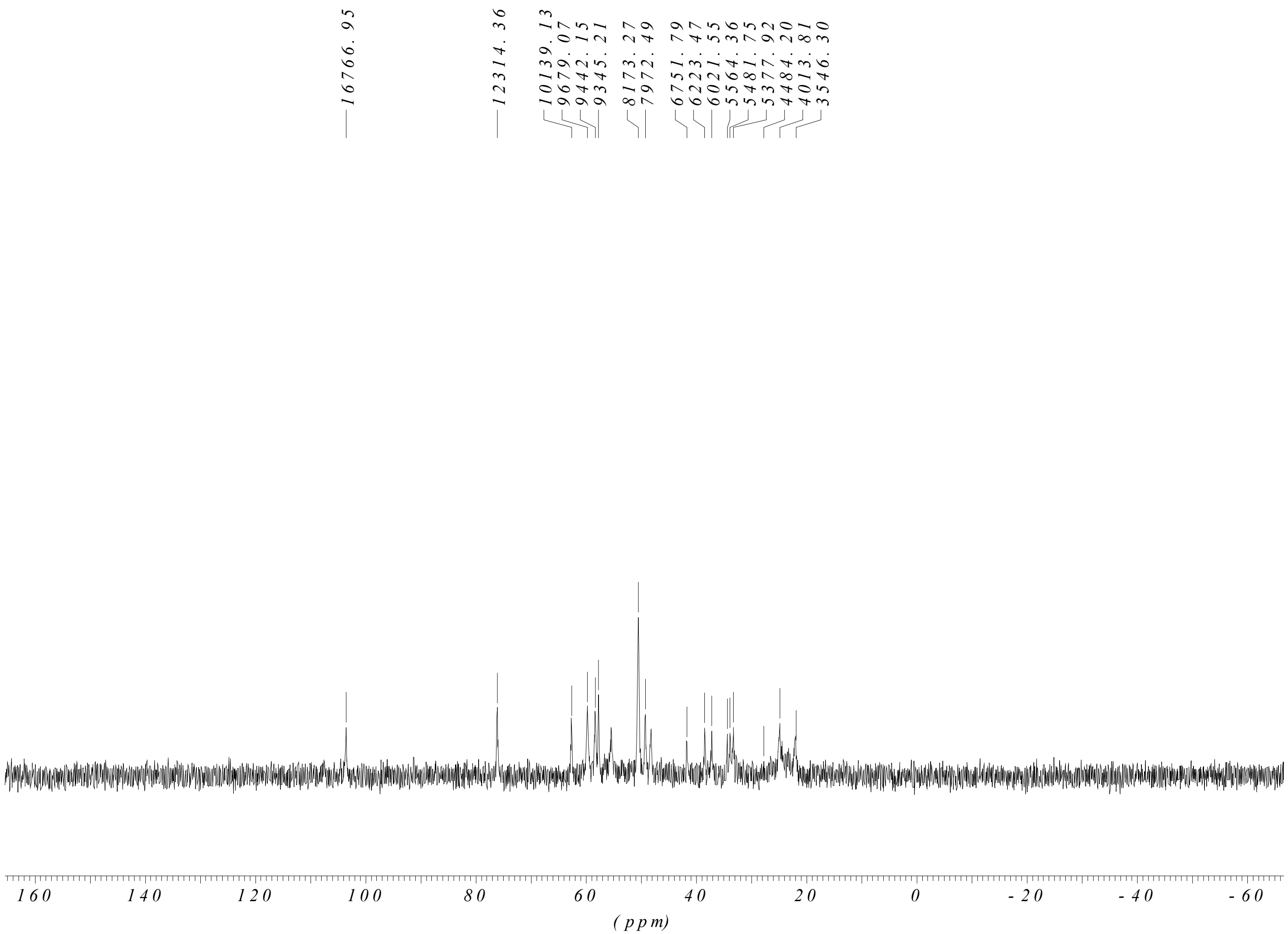

S2.3.6 $^{31}P_{dec}$ NMR spectrum ($CDCl_3$) of a mixture of dioctylphosphine and trioctylphosphine oxides **3d,e** (Table 2, experiment No. 4).

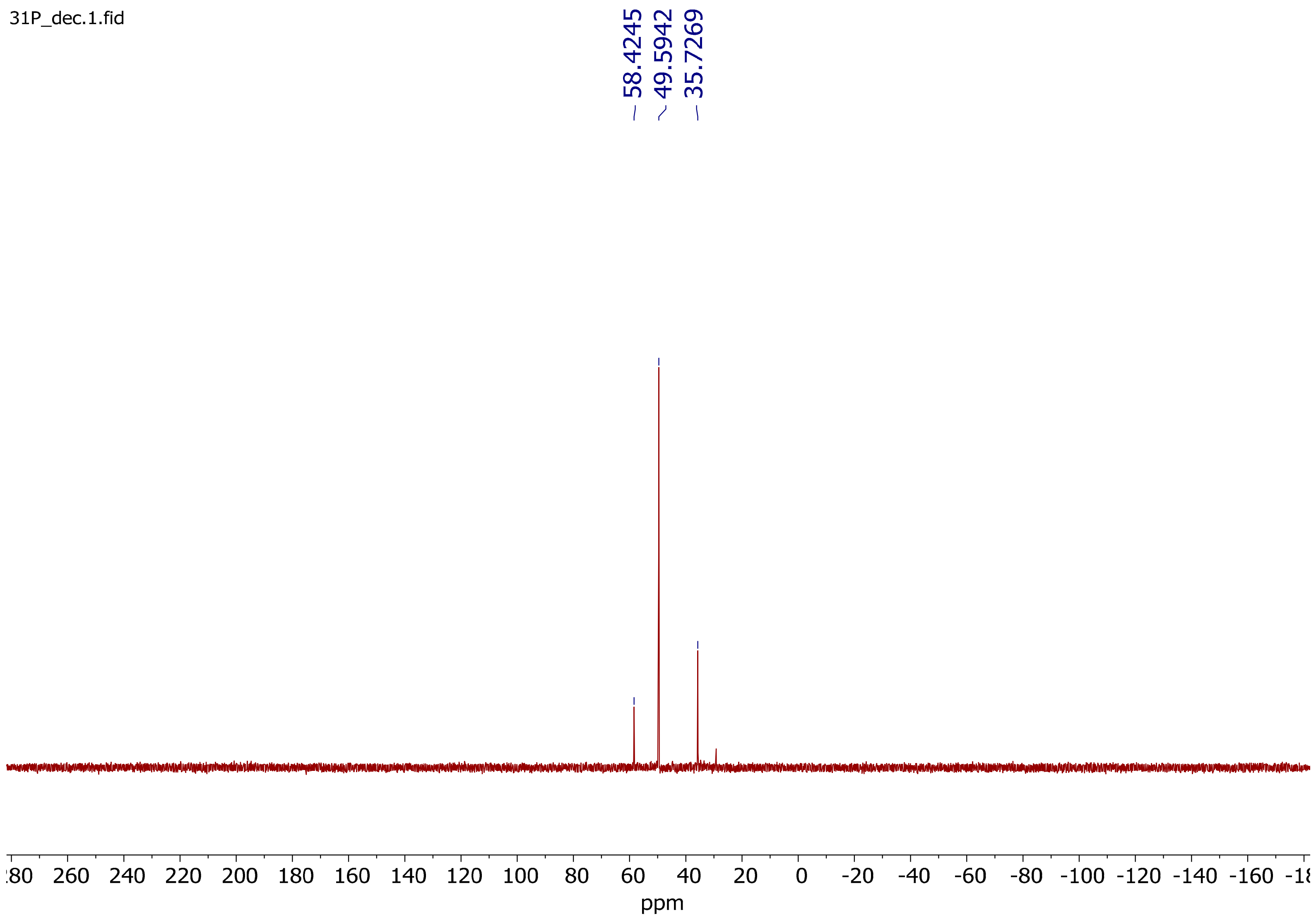

S2.3.7 $^{31}P_{cop}$ NMR spectrum ($CDCl_3$) of a mixture of dioctylphosphine and trioctylphosphine oxides **3d,e** (Table 2, experiment No. 4).

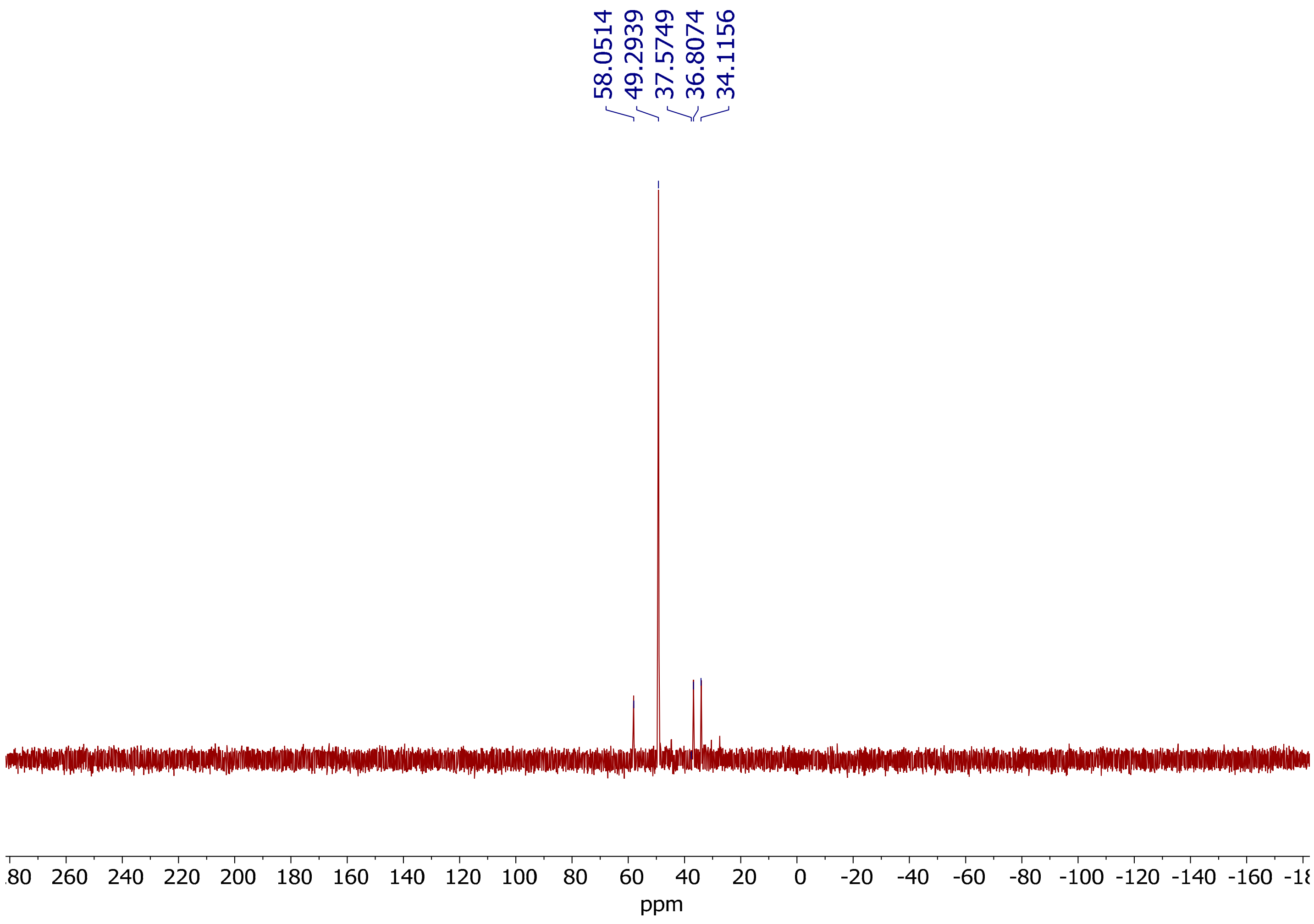

Supplement: Supplementary file 1 [file materials-16-03394-s001.zip › materials-2319284-supplementary.pdf]
